# GUANIN: an all-in-one GUi-driven analyzer for NanoString interactive normalization

**DOI:** 10.1093/bioinformatics/btae462

**Published:** 2024-07-25

**Authors:** Julián Montoto-Louzao, Alberto Gómez-Carballa, Xabier Bello, Jacobo Pardo-Seco, Alba Camino-Mera, Sandra Viz-Lasheras, María J Martín, Federico Martinón-Torres, Antonio Salas

**Affiliations:** Unidade de Xenética, Instituto de Ciencias Forenses, Facultade de Medicina, Universidade de Santiago de Compostela, and Genética de Poblaciones en Biomedicina (GenPoB) Research Group, Instituto de Investigación Sanitaria (IDIS), Hospital Clínico Universitario de Santiago (SERGAS), 15706, Santiago de Compostela, Spain; Genetics, Vaccines and Infections Research Group (GENVIP), Instituto de Investigación Sanitaria de Santiago, Santigo de Compostela, 15706, Spain; Centro de Investigación Biomédica en Red de Enfermedades Respiratorias (CIBER-ES), Madrid, 28029, Spain; Unidade de Xenética, Instituto de Ciencias Forenses, Facultade de Medicina, Universidade de Santiago de Compostela, and Genética de Poblaciones en Biomedicina (GenPoB) Research Group, Instituto de Investigación Sanitaria (IDIS), Hospital Clínico Universitario de Santiago (SERGAS), 15706, Santiago de Compostela, Spain; Genetics, Vaccines and Infections Research Group (GENVIP), Instituto de Investigación Sanitaria de Santiago, Santigo de Compostela, 15706, Spain; Centro de Investigación Biomédica en Red de Enfermedades Respiratorias (CIBER-ES), Madrid, 28029, Spain; Unidade de Xenética, Instituto de Ciencias Forenses, Facultade de Medicina, Universidade de Santiago de Compostela, and Genética de Poblaciones en Biomedicina (GenPoB) Research Group, Instituto de Investigación Sanitaria (IDIS), Hospital Clínico Universitario de Santiago (SERGAS), 15706, Santiago de Compostela, Spain; Genetics, Vaccines and Infections Research Group (GENVIP), Instituto de Investigación Sanitaria de Santiago, Santigo de Compostela, 15706, Spain; Centro de Investigación Biomédica en Red de Enfermedades Respiratorias (CIBER-ES), Madrid, 28029, Spain; Unidade de Xenética, Instituto de Ciencias Forenses, Facultade de Medicina, Universidade de Santiago de Compostela, and Genética de Poblaciones en Biomedicina (GenPoB) Research Group, Instituto de Investigación Sanitaria (IDIS), Hospital Clínico Universitario de Santiago (SERGAS), 15706, Santiago de Compostela, Spain; Genetics, Vaccines and Infections Research Group (GENVIP), Instituto de Investigación Sanitaria de Santiago, Santigo de Compostela, 15706, Spain; Centro de Investigación Biomédica en Red de Enfermedades Respiratorias (CIBER-ES), Madrid, 28029, Spain; Unidade de Xenética, Instituto de Ciencias Forenses, Facultade de Medicina, Universidade de Santiago de Compostela, and Genética de Poblaciones en Biomedicina (GenPoB) Research Group, Instituto de Investigación Sanitaria (IDIS), Hospital Clínico Universitario de Santiago (SERGAS), 15706, Santiago de Compostela, Spain; Genetics, Vaccines and Infections Research Group (GENVIP), Instituto de Investigación Sanitaria de Santiago, Santigo de Compostela, 15706, Spain; Unidade de Xenética, Instituto de Ciencias Forenses, Facultade de Medicina, Universidade de Santiago de Compostela, and Genética de Poblaciones en Biomedicina (GenPoB) Research Group, Instituto de Investigación Sanitaria (IDIS), Hospital Clínico Universitario de Santiago (SERGAS), 15706, Santiago de Compostela, Spain; Genetics, Vaccines and Infections Research Group (GENVIP), Instituto de Investigación Sanitaria de Santiago, Santigo de Compostela, 15706, Spain; CITIC, Computer Architecture Group, Universidade da Coruña, Facultad de Informática, 15071, A Coruña, Spain; Genetics, Vaccines and Infections Research Group (GENVIP), Instituto de Investigación Sanitaria de Santiago, Santigo de Compostela, 15706, Spain; Centro de Investigación Biomédica en Red de Enfermedades Respiratorias (CIBER-ES), Madrid, 28029, Spain; Translational Pediatrics and Infectious Diseases, Department of Pediatrics, Hospital Clínico Universitario de Santiago de Compostela, Santiago de Compostela, Choupana s/n, Santiago de Compostela, 15706, Spain; Unidade de Xenética, Instituto de Ciencias Forenses, Facultade de Medicina, Universidade de Santiago de Compostela, and Genética de Poblaciones en Biomedicina (GenPoB) Research Group, Instituto de Investigación Sanitaria (IDIS), Hospital Clínico Universitario de Santiago (SERGAS), 15706, Santiago de Compostela, Spain; Genetics, Vaccines and Infections Research Group (GENVIP), Instituto de Investigación Sanitaria de Santiago, Santigo de Compostela, 15706, Spain; Centro de Investigación Biomédica en Red de Enfermedades Respiratorias (CIBER-ES), Madrid, 28029, Spain

## Abstract

**Summary:**

Most tools for normalizing NanoString gene expression data, apart from the default NanoString nCounter software, are R packages that focus on technical normalization and lack configurable parameters. However, content normalization is the most sensitive, experiment-specific, and relevant step to preprocess NanoString data. Currently this step requires the use of multiple tools and a deep understanding of data management by the researcher. We present GUANIN, a comprehensive normalization tool that integrates both new and well-established methods, offering a wide variety of options to introduce, filter, choose, and evaluate reference genes for content normalization. GUANIN allows the introduction of genes from an endogenous subset as reference genes, addressing housekeeping-related selection problems. It performs a specific and straightforward normalization approach for each experiment, using a wide variety of parameters with suggested default values. GUANIN provides a large number of informative output files that enable the iterative refinement of the normalization process. In terms of normalization, GUANIN matches or outperforms other available methods. Importantly, it allows researchers to interact comprehensively with the data preprocessing step without programming knowledge, thanks to its easy-to-use Graphical User Interface (GUI).

**Availability and implementation:**

GUANIN can be installed with pip install GUANIN and it is available at https://pypi.org/project/guanin/. Source code, documentation, and case studies are available at https://github.com/julimontoto/guanin under the GPLv3 license.

## 1 Introduction

NanoString nCounter (Geiss *et al.* 2008) is a molecular barcoding platform for direct multiplexed quantification of RNA molecules in biological samples. It reports actual counts of sequences of interest through image analysis. As no amplification is needed, it avoids potential bias introduced by reverse transcription, striking a balance between limitations of RNA-seq and microarrays. Due to its robust performance, NanoString is mainly used in experiments involving low quality samples, and/or tissue samples for the identification of nucleic acid presence, where proper quality control (QC) and normalization are crucial to maintain the accuracy of the experiments (Gagnon-Bartsch and Speed 2012).

The gene expression platform offers nSolver, a GUI software freely available at (https://nanostring.com/products/ncounter-analysis-system/nsolver-advanced-analysis-software/). nSolver addresses background correction, positive control (technical) normalization, and housekeeping normalization. Current state-of-the-art tools have specialized in improving normalization in different ways, including: (i) Technical normalization: NanoStringNorm (Waggott *et al.* 2012) and NanoStringDiff (Wang *et al.* 2016); (ii) Effective unwanted variation removal: RCRNorm (Jia *et al.* 2019), RUV-III (Molania *et al.* 2019), and an approach utilizing an extending iterative framework of RUVSeq (Bhattacharya *et al.* 2021); (iii) Up-scalability and automatization: nf-core/nanostring (Peltzer *et al.* 2024); and (iv) Accessibility and data visualization: NanoTube (Class *et al.* 2023) and NACHO (Canouil *et al.* 2020). Therefore, a comprehensive normalization analysis would involve the use of several of these packages, requiring in-depth knowledge of the field, programming skills, and tedious data management. Moreover, in previous approaches, the utilities for content normalization are limited and lack comprehensive configuration options, including the selection, refining, and evaluation of candidate reference genes, among other features.

Wide parametrization is key to accurate preprocessing, however, current tools are not as configurable as needed for specific experiments, often resulting in dead ends and/or the development of analyses with suboptimal normalization. To offer a comprehensive, easy-to-use, interactive pipeline for NanoString data preprocessing, we created GUANIN, a user friendly, widely configurable, and cutting edge updated tool for NanoString interactive normalization.

## 2 Materials and methods

**Figure 1.**
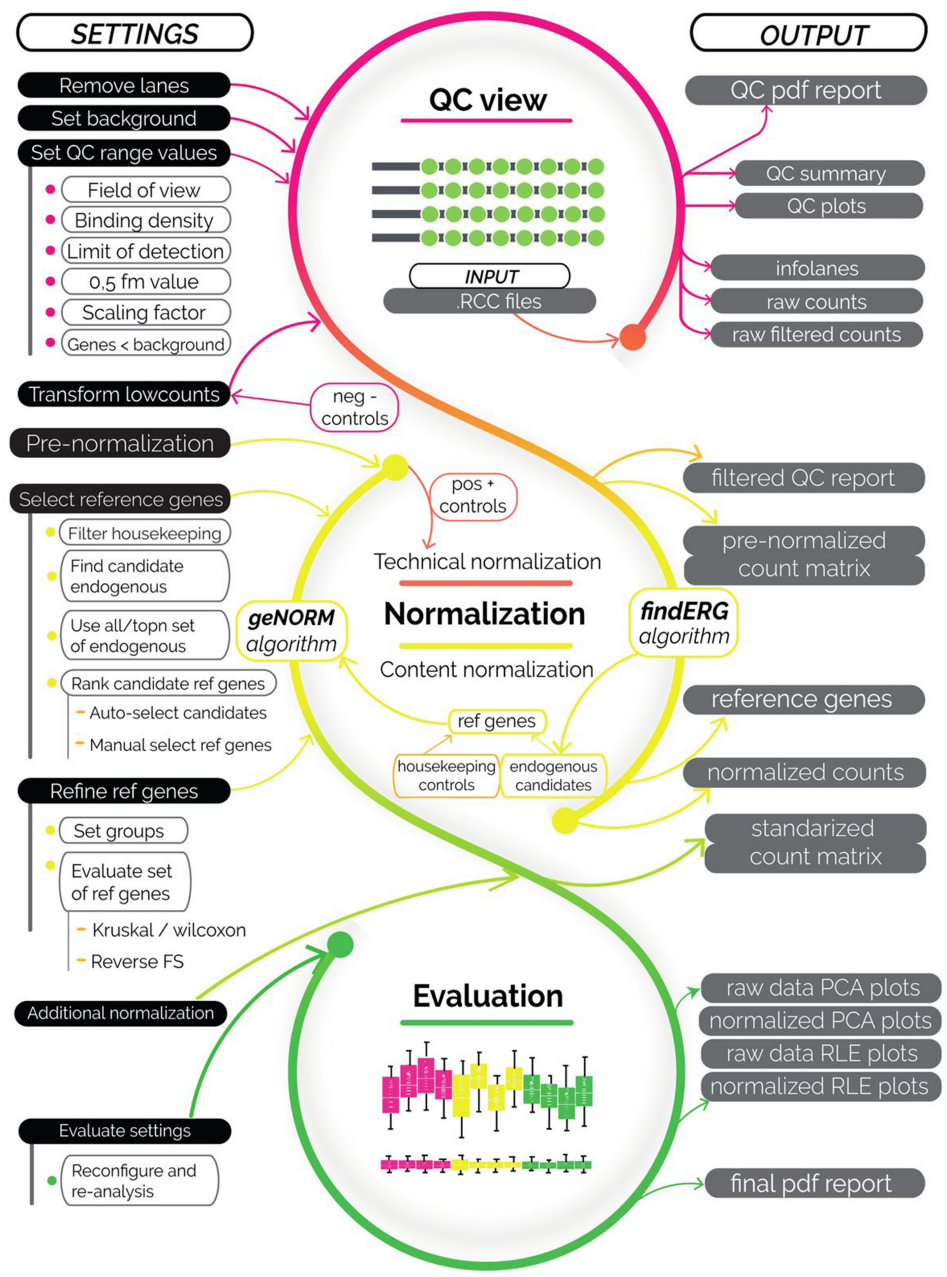
Main workflow of GUANIN with input settings on the left and output on the right.

GUANIN workflow (Fig. 1) starts with load and inspection of input data, continues with background correction, technical normalization (assessing experimental variations) and content normalization (assessing biological variability). Additionally, it offers the possibility to perform additional formatting of output data and evaluation of normalization process. For detailed information about all GUANIN features see Supplementary Methods.

### 2.1 Step 1: Loading RCC files and generating a QC report

GUANIN starts data processing from Reporter Code Count (RCC) files and a recommended metadata file. Right after loading input files, the first analysis reports the inherent information from the experiment needed to perform adequate normalization through both a .pdf and a .html QC report. From here, QC parameters such as background, lanes to remove, or QC acceptation ranges can be recursively modified until an optimal QC status is achieved. For this, GUANIN implements several methods of background calculation, a new implementation to select alternative negative control genes, different methods for background correction and tweakable ranges for every QC parameter (counts below background, binding density, field of view, linearity, and scaling factor). This process can be supported by the visualization of the .pdf QC report generated automatically after loading the files.

### 2.2 Step 2: Normalization

Based on the cutting-edge methodologies for NanoString Normalization, GUANIN pipeline can perform normalization through two main ways: traditional scaling factors (involving technical and content normalization) or removing unwanted variation.

- *Scaling factor normalization*. In contrast to the nSolver pipeline, and as an exclusive feature, GUANIN workflow allows to perform technical normalization before background correction, as it has shown improved normalization results (Lin *et al.* 2016). Scaling factor can be calculated from various sample-derived measures or applying a regression model to the data, in the style of RCRNorm (Jia *et al.* 2019), as a single scaling factor does not effectively normalize both lowly and highly expressed genes (Hafemeister and Satija 2019). For content normalization, a set of reference genes needs to be chosen. In addition to default housekeeping genes, and unlike other tools, GUANIN allows selecting candidate reference genes from among endogenous, as it is a common issue that housekeeping genes are not suitable for some experiments. We utilized ERGene (Zeng *et al.* 2020), a Python library for screening endogenous reference genes, to implement this utility. The candidate reference genes, including n selected endogenous genes and the panel of housekeeping genes, are evaluated using a geNORM-based algorithm (Vandesompele *et al.* 2002, Zhong 2019) to select n genes for driving content normalization. In addition, a modification to this algorithm was implemented, offering the option to weight the geNORM selection according to the relevance of the gene used as reference (Supplementary Methods). Subsequently, the candidate reference genes are filtered or flagged by a three-way group-driven differential expression analysis among groups, employing the Kruskal-Walllis (Kruskal and Wallis 1952), Wilcoxon rank sum test (Wilcoxon 1992), and a reverse sequential feature-selection method (Gelsema and Kanal 2014) that considers the combined effect of several candidate genes. Alternatively, other options, such as manual selection of reference genes using most expressed endogenous or all endogenous expression, are also available to the researcher. Once content normalization is performed, additional normalization, such as standardization, is also available.
- *RUVg norm algorithm*. GUANIN implements a python port of the efficient RUVg algorithm (Risso, *et al.* 2014), including k value easy selection and the possibility to implement pyDESEQ2’s (Love *et al.* 2014, Muzellec *et al.* 2023), median-of-ratios pre-normalization method. Selection and refining of candidate reference genes can be also applied to RUVg normalization.

### 2.3 Step 3: Evaluation of normalization results

The normalization results are evaluated through computation of the interquartile range and graphical analysis using Relative Log Expression (RLE) plots (Gandolfo and Speed 2018), which compare the raw data with the normalization results (Supplementary Methods). Additionally, GUANIN allows the visualization of Principal Component Analysis (PCA) plots comparing batch effect on raw data and condition-explained effect on normalized data. These plots provide a straightforward visualization of the results, making possible an easy re-parametrization and re-run of some steps of the process.

## 3 Results

The main aim of GUANIN is to provide a flexible and adaptable parametrization to perform the normalization that best suits the experiment. To evaluate GUANIN, we have examined three studies, including one in-house dataset of a COVID-19 study (GEO accession number: GSE183071) and two published datasets (GSE160208, GSE108395) that can assess several standard casuistic issues when analyzing NanoString data. GUANIN obtains good RLE plots and provides accurate normalized data for all the three studies. See Supplementary Methods for more details.

Furthermore, drawing upon our experience as seasoned users of NanoString data normalization, we have designed GUANIN with a user-friendly interface and broad experiment compatibility in mind. Specifically, its features include:

- Multiplatform, user-friendly, Python based.
- Wide compatibility with different editions of RCC format, column names, and gene identifiers, capable of preprocess miRNA and RNA experiments by default.
- Optional visualization of results and QC for every step.
- A wide range of QC and normalization options, including the best up-to-date algorithms and several new improvements.
- The most thorough, adaptable, and comprehensive content normalization, which includes selection, ponderation, refinement and validation of housekeeping and endogenous candidate genes.
- In-built evaluation of normalization and recursive and accessible modification of the normalization pipeline.

Thanks to all these characteristics, GUANIN enables to easily detect and manage normalization and/or QC problems within the experiment. In addition, while other software packages might be unable to address these issues, GUANIN allows adapting the whole normalization process to provide accurate normalized data.

## 4 Discussion

Assuming pre-built-in housekeeping genes from NanoString will function properly as reference genes for an experiment is naive, especially when dealing with different tissues and metabolic processes. Therefore, including endogenous genes as candidate reference genes proves to be a valuable option. The geNorm intelligent evaluation and selection process implemented into GUANIN includes at least three endogenous genes in best 6-gene selection to be used as reference genes.

RUVg normalization is one of the most trusted methods for NanoString normalization, but it can be problematic when there is only a small number of negative controls (Jia *et al.* 2019). Having the ability to detect, address and use the new algorithms (i.e. election of alternative negative controls) and configurations introduced in GUANIN can be key to be able to perform the best pipeline for the characteristics of the experiment, getting over usual experimental hassles.

Perfect normalization results are usually not feasible, as ground truth is not reachable. For this, the RLE or PCA plots provided by GUANIN allow the user to identify the best method to fit their data. Centered and narrow RLE plots indicate variation removal, although some biological information might also be lost. Grouped PCAs by condition, rather than by batch, are expected to be equivalent of a proper normalization process, but batch effect can still persist in the data.

Being able to try amongst huge combination of normalization options and to refine and compare the effect on the output data of this parametrization is key for an adequate normalization. In fact, GUANIN’s results are particularly promising when the analysis can be refined, which is frequently the case in exploratory or confirmatory studies. It can address issues such as poor housekeeping performance, poor negative control, low general expression, and suboptimal experiment design. This is common, as most NanoString panels are preset, and poor QC is often encountered. Thus, having the ability to conduct a thorough analysis and adapt preprocessing to each experiment without an in-depth knowledge of R is a useful advantage for health researchers.

While no other tool provides as wide interactive parametrization as GUANIN does, NACHO can be useful for a smooth alternative visualization, and RUV-III offers excellent results in removing unwanted variation, although it requires technical replicates, which in practice is very infrequent. GUANIN offers excellent results, which, combined with its wide flexibility and user-friendly interface, make it a convenient preprocessing tool for clinical scientists seeking a fast, reliable, and comprehensive method to preprocess their data and obtain visual reports of the results. It can be also a useful tool for experienced scientists with programming experience, as it allows for an easy transition from RCCs to normalized data, provides a command–line interface (CLI) that enables its use on servers, or the possibility to import its functions on Python scripting, facilitating the creation of custom pipelines.

## Supplementary Material

btae462_Supplementary_Data
