# Supplementary material for: GUANIN: an all-in-one GUi-driven analyzer for NanoString interactive normalization
Source: Bioinformatics. 2024 Jul 25;40(8):btae462. doi: 10.1093/bioinformatics/btae462 (PMC11333565; doi:10.1093/bioinformatics/btae462)
Supplement: btae462_Supplementary_Data [file btae462_supplementary_data.docx]

Supplementary information


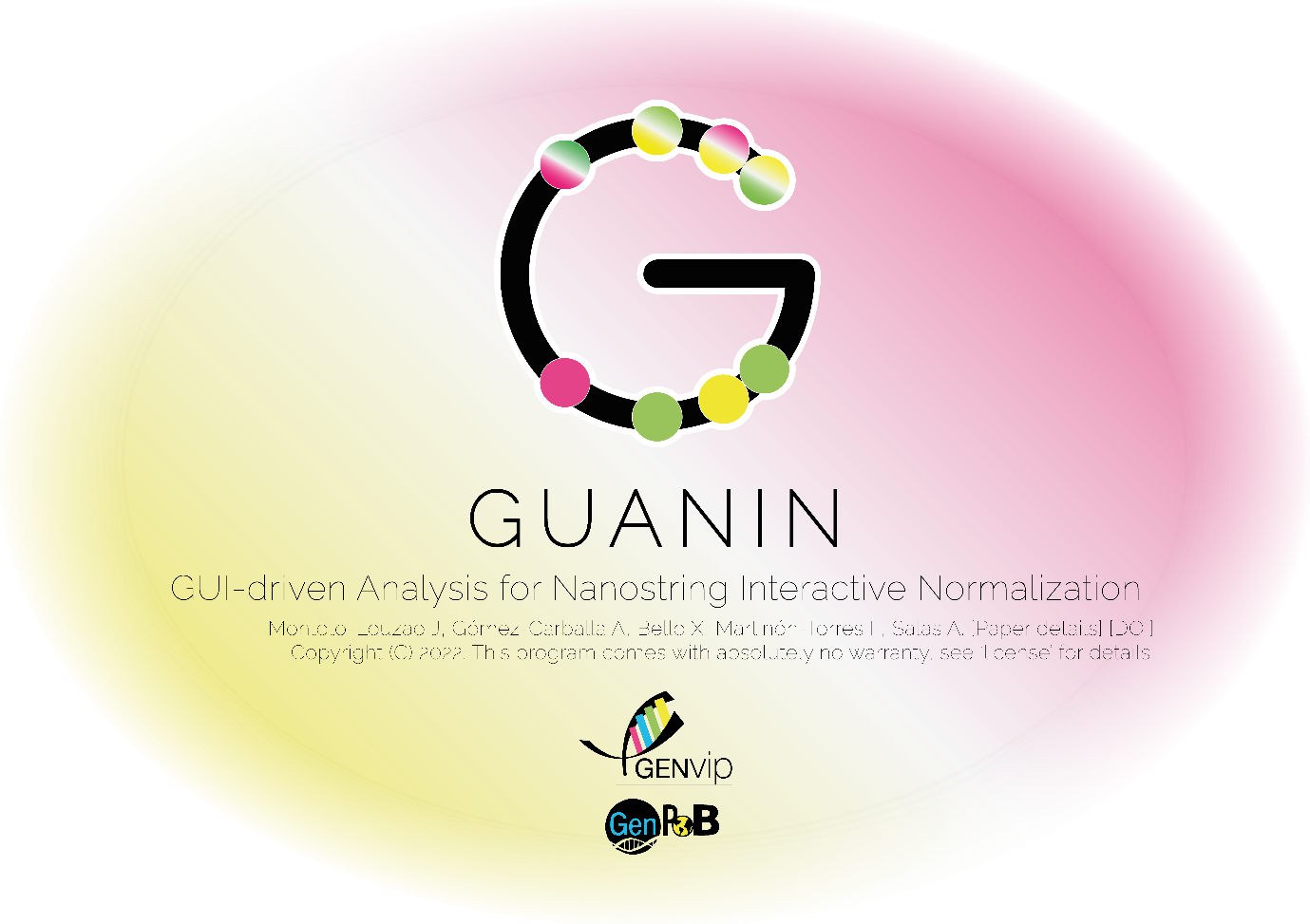


Based on GUANIN version 1.2.10: 20/05/2024

User guide version: 1.0: 20/05/2024

Please cite:

**1. WORKFLOW DESCRIPTION**


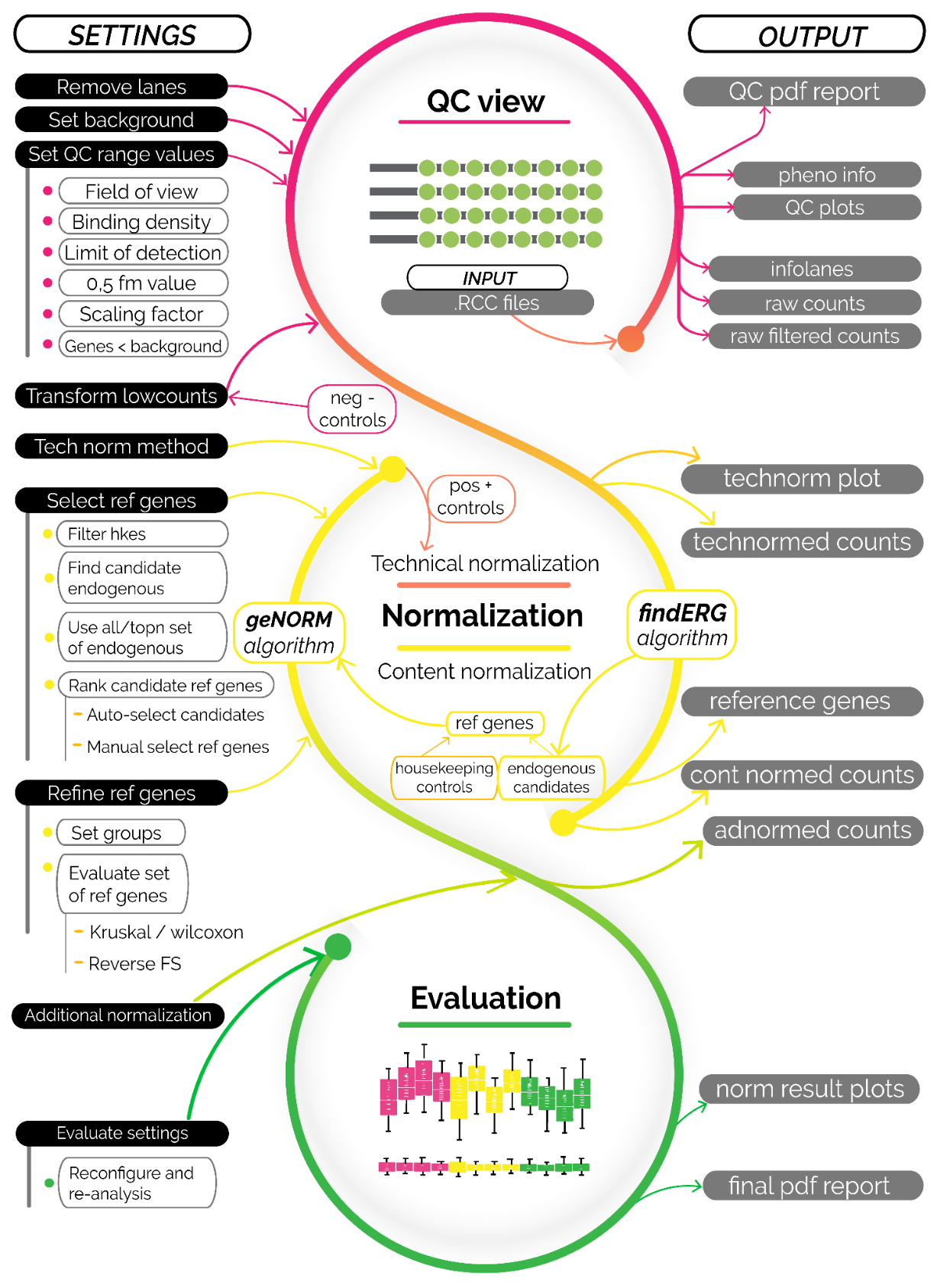
Figure 1 shows GUANIN workflow. It starts with load and inspection of input data (.RCC files), continues with technical normalization (assessing experimental variations), background correction and content normalization (assessing biological variability). Additionally, it offers the possibility of performing additional normalization, formatting output data, and evaluating the normalization process.

**Figure 1: Simplified GUANIN workflow.**

**1.1 INSTALLATION**

Having pip and Python 3.8 or higher previously installed, installing GUANIN in any OS should be as easy as run the following command:

$ pip install guanin

For installation details on every OS, see README.md and INSTALL.txt on github.com/julimontoto/guanin.

The installation includes the four example dataset described in Section 3 located within the GUANIN installing folder.

**1.2 GRAPHICAL USER INTERFACE OVERVIEW**


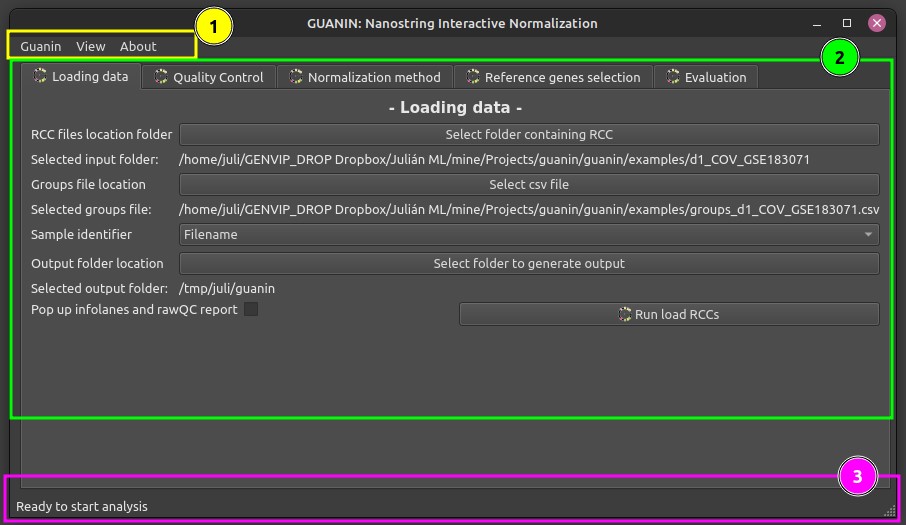


**Figure 2: GUANIN user interface.**

Figure 2 shows the main window of the GUANIN graphical user interface. It is divided into 3 sections:

1. General GUANIN options.

1.a. Guanin.

1.b. View: straightforward access to different resources included in the software: user guide, analysis logs, generated pdf report (if already generated), QC summary (if already generated) and output folder.

1.c. About.

2. Normalization steps tabs and parameters. Usually, users can run GUANIN several times tweaking the parameters from each tab one or more times, based on the reports and files generated, until best configuration for the experiment is found.

3. Status bar: shows information about running process. Note that during the execution of the program the window might become blocked for a few seconds when svg files are being generated (optional), particularly if high number of samples are uploaded. The user can check the running state of GUANIN in the status bar.

**1.3 INPUT DATA**

The type of data required to run GUANIN are raw .RCC (Reporter Code Count) files. The use of preprocessed data as input is not recommended. Analysis starts with the selection of the folder containing the .RCC files (Fig 3. Point 1).

A metadata file in CSV (Comma Separated Value) format containing samples groups info can be provided to refine content normalization (Fig 3. Point 2). This step is not mandatory, but it is recommended to improve normalization results. It can be created by the user using a text editor or spreadsheet software, such as Excel. It should contain 2/3 columns: “SAMPLE” that includes samples IDs and needs to match with sample identification in the .RCC files (sample ID or file name), “GROUP”, the comparison groups, and optionally “BATCH”, used to visualize batch effect removal. The “GROUP” column should include the phenotype or condition of interest for each sample, and should not contain information about batches or replicates.


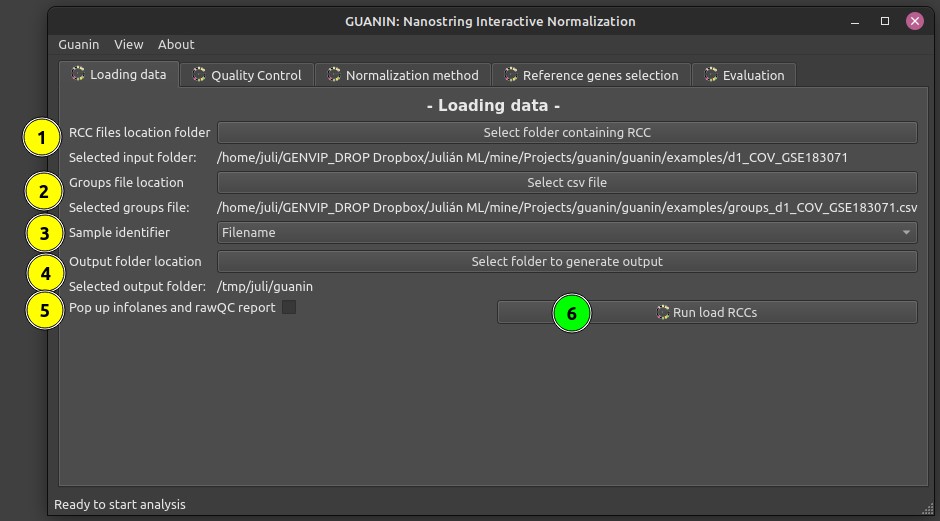


**Figure 3: GUANIN 'loading data' tab.**

Make sure to successfully select your groups file. If you are not providing a groups file, click “Select csv file” and close the window, so you see the dialog: “No groups file selected”.

Four ready-to-use example datasets downloaded from GEO (Gene Expression Omnibus) including .RCC files and metadata groups CSV files are provided with GUANIN, located in separate folders in the same GUANIN installation folder (which may depend on your OS and configuration):

- GSE183071 – Blood study of gene expression profiling on the nasal epithelium in COVID-19 severity
- GSE160208 – Gene expression in the brain of sporadic Creutzfeldt-Jakob disease patients (CJD), and normal controls (CT)
- GSE108395 – Differential expression analysis of miRNAs expressed in Huntington’s disease.
- GSE126549 – Characterizing changes in miRNA signatures in fallopian-tube derived mouse cancer models.

“Filename” is the default parameter for the sample identifier as it is foolproof (Fig 3. Point 3), but in case of a sample name has been included in the “sample ID” field from the .RCC files and this information is reliable (no duplicates, etc), then the use of “sample ID” as sample identifier is recommended. Spaces within SampleIDs (or filenameIDs) are not allowed (see Section 1.4).

Output folder is set by default in the system’s temp folder, but it can be modified by the user (Fig 3. Point 4). It is recommended to open output folder in a file explorer in order to check images, reports and tables generated on the go by GUANIN, as back and forth parametrization can be done using this information. This can be easily accessed by clicking in the menu bar “View” → “Open output folder”.

Information about lanes and raw QC (Quality Control) report can be set to automatically open these files in the default web browser (Fig 3. Point 5) right after loading the .RCC files (Fig 3. Point 6). This information can be also checked by accessing the files in the selected output folder.

In this step, the following output files and reports are generated:

count matrix of raw counts (csv)

rawsummary (html and csv)

rawinfolanes (html and csv)

raw QC inspection report (pdf)


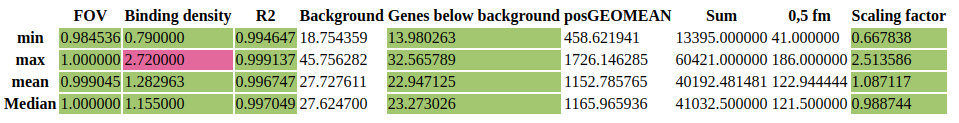


**Figure 4: html raw summary report.**

Figure 4 shows an example of the rawsummary.html file. It contains minimum, maximum, mean, and median values for:

- Field of view (FOV): proportion of imaging sections that are successfully imaged.
- Binding density: level of image saturation, min and max number of optical features per square micron for each lane.
- R^2^: linearity value related the efficiency of the hybridization. High regression correlation. between the known positive control’s concentration and the resulting counts from them.
- Background (default): calculated reference value from the negative controls. By default, mean + 2*std.
- Genes below background: % of genes with lower expression than the background.
- *PosGEOMEAN*: positive geometric mean calculated from positive controls. Default value to infer the scaling factor (technical normalization).
- Sum: summation of positive controls, alternative value to infer the scaling factor.
- 0,5fm: counts for the positive control with 0,5fM concentration.
- Scaling factor: ratio of positive controls expression for the lane and positive controls mean expression used to perform technical normalization.


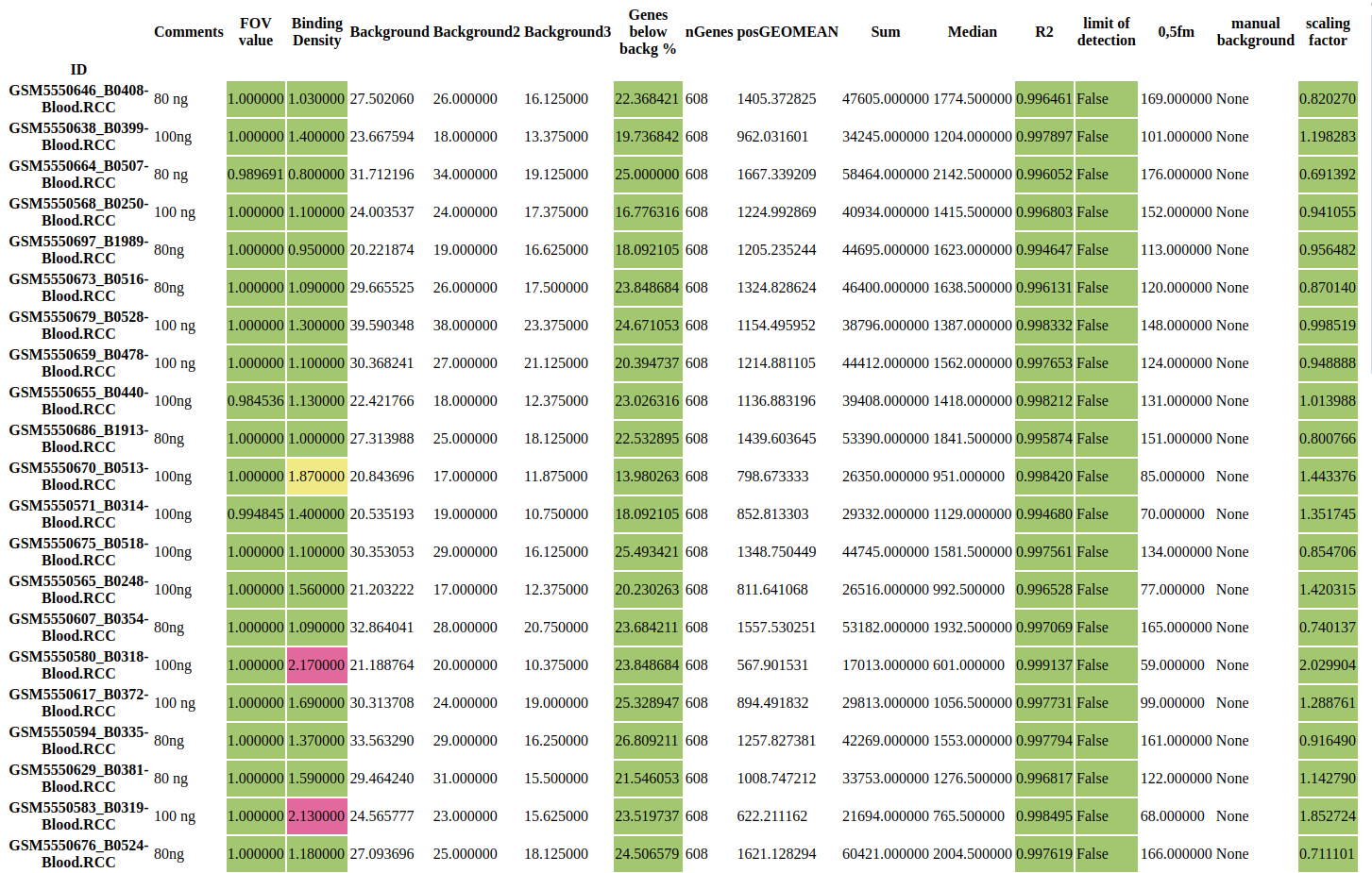


**Figure 5: html raw infolanes report.**

Figure 5 shows an example of the rawinfolanes.html file. It contains initial QC information per sample. Some additional features are included in this report:

- Background2: Maximum value from negative controls.
- Background3: Mean value from negative controls.
- Limit of detection: detection of 0,5 fM concentration positive control probe. True would mean that the 0.5fM counts are below the chosen background level.

For more information about QC values see https://nanostring.com/wp-content/uploads/Gene_Expression_Data_Analysis_Guidelines.pdf


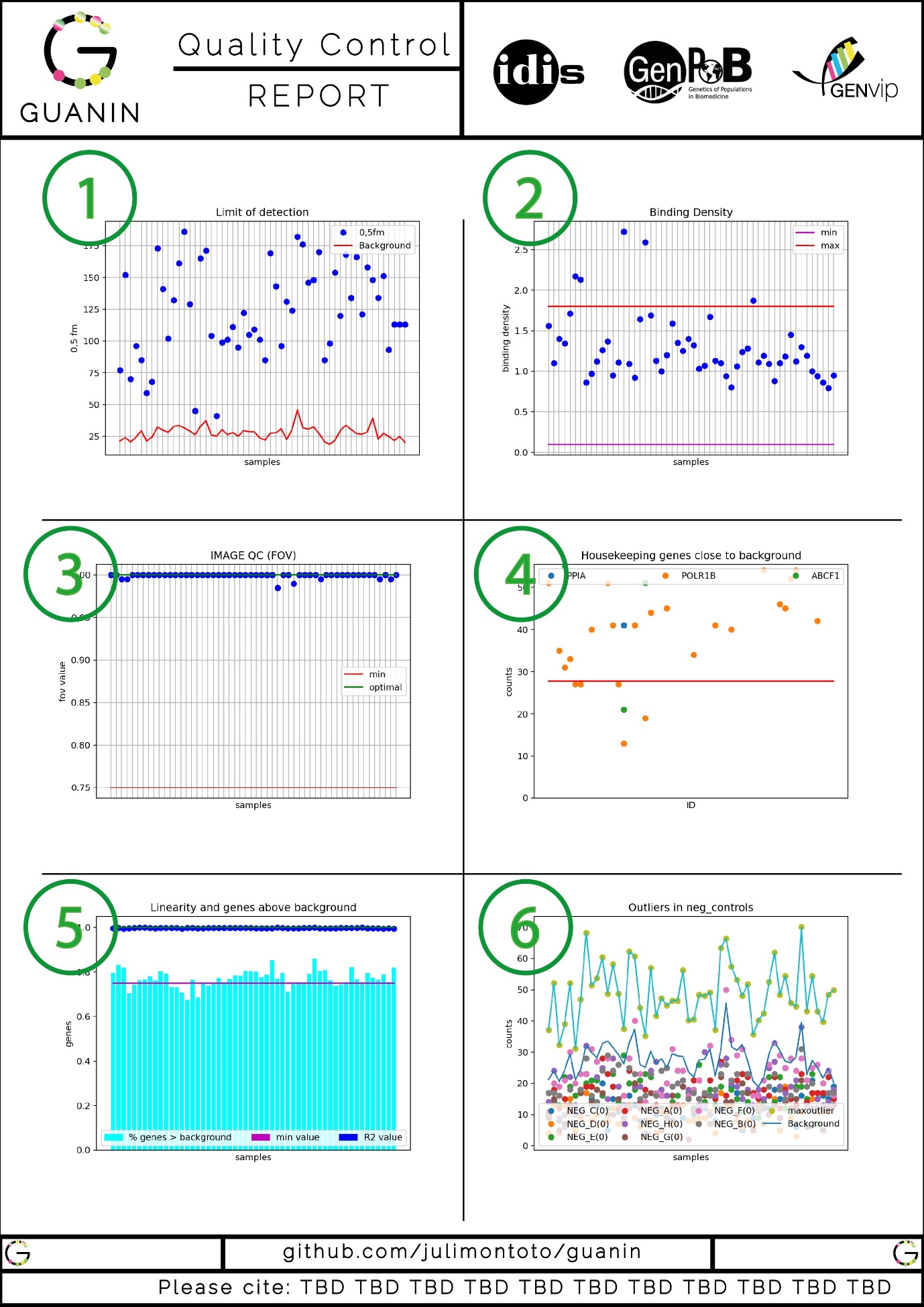


**Figure 6: pdf QC report**.

Figure 6 shows an example of the QC report. The information is displayed using 6 different plots:

1. Positive control E probe (0.5fM) counts for each sample and the background value per sample.

2. Image saturation, showing values below, in between and above the accepted values range. Default range tops at 1.8 (as NanoString recommends for FLEX/MAX) but in case of SPRINT max value can be set at 2.25, see https://nanostring.com/wp-content/uploads/Gene_Expression_Data_Analysis_Guidelines.pdf

3. Field of views (FOV), they are expected to be close to 1 and never below the minimum accepted value.

4. Low expressed housekeeping genes expression values per sample. We can check if there are samples with housekeeping genes expressing bellow the background. Detailed info about expression values for the housekeeping genes can be found at the output folder ‘otherfiles/dfhkecount.csv’.

5. Linearity value (R2), it is expected to be close to 1 (see green line), as expected and observed concentration of positive controls should tend to 1. % of genes above background is ideally close to 100% too. So, R2 dots for each sample and bars above min value pass QC.

6. Outliers in negative controls. All values below *maxoutlier* (maximum value to be considered an outlier, calculated as three times the mean of negative controls for that sample) are adequate. Values between background and *maxoutlier* can be considered a warning (eg: NEG_F). Values below background are expected. Higher values for a subset of negative controls could be the reason to disregard these negative controls and/or use an alternative subset of negative controls.

Note: Aside from the pdf report, all higher resolution vector images (.svg files) generated can be found at output/images/vectors directory.

**Note about control genes:**

GUANIN follows a Nanostring-based definition for control genes, as follows:

- Negative controls: probes that recognize synthetic mRNA targets not included in the CodeSet. They are used to establish the background signal.
- Positive controls: probes that recognize synthetic mRNA targets included in the CodeSet at specified concentrations, used to confirm linear response to input amounts, and confirm that low input signal is above background and to infer scaling factor for technical normalization.
- Ligation positive controls: probes that recognize synthetic miRNA targets included in the Sample Preparation Kit. Ligation positive controls monitor ligation efficiency, independent of the miRNAs in the sample.
- Ligation negative controls: probes that recognize synthetic miRNA targets not included in the Sample Preparation Kit (no miRNA target). Ligation negative controls monitor non-specific ligation.
- Housekeeping genes (referred exclusively to those pre-defined in the Nanostring panel): probes for genes with expected high and stable expression used for content normalization.
- Reference genes: A subset of filtered housekeeping genes and/or other candidate reference genes (from stably expressed endogenous, for example) used for normalization.

**1.4 QUALITY CONTROL ANALYSIS**


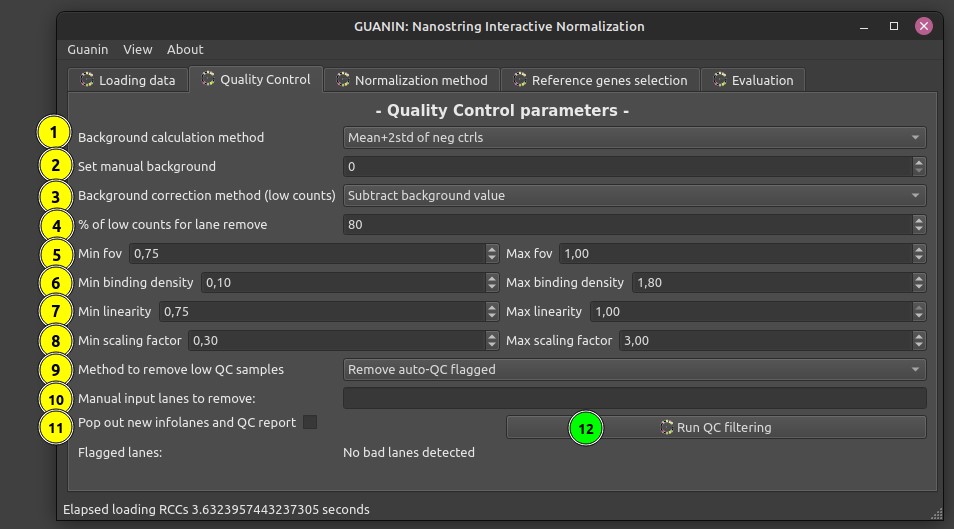


**Figure 7: GUANIN 'Quality Control' tab.**

GUANIN allows performing different QC analyses. Default values are based on NanoString guidelines. QC specific parameters information can be better understood by reading NanoString Gene Expression Data Analysis Guidelines or see Chilimoniuk et al. (2024).

It is recommended to use the default values for the first executions of GUANIN. The user can customize the parameters after running GUANIN back and forth and checking the reports and tables generated by the software.

Figure 7 shows the configurable options:

- *Background determination (Fig 7. Point 1)*:

Spike-in negative controls are included in NanoString panels to set a threshold for non-expressed genes: the background. This threshold can be restrictive depending on the characteristics of the experiment. GUANIN default background setting is conservative, and it is calculated as the mean of the negative controls + twice the standard deviation. Other options can be selected by the user, such as the maximum counts value of negative controls or the mean of negative controls. GUANIN implements a new alternative method to select the background (see Section 2.1), useful in case there is a problem with the Spike-in negative controls (i.e: high number of counts). In this case, an alternative method for background calculation can be selected, using low-expressed probes among the endogenous genes. Additionally, background can be set manually (Fig 7. Point 2).

- *Background correction (Fig 7. Point 3)*:

Once background is set, there are several options to handle values below background threshold (low counts):

1. Set as background: Sets all counts values below the background threshold as equal to background.
2. Subtract background: subtract the background value from all gene counts of the dataset, assigning a value of 0 to those genes expressed equally or lower than the background threshold (default).
3. Skip: Ignore background correction (no background correction).

- *Sample QC*:

Samples with QC issues can be a) flagged or b) removed from the analysis. Technical parameters considered for QC analysis are the following (see NanoString Gene Expression Data Analysis Guidelines):

1. High % of genes below background (Fig 7. Point 4): lot of genes expressing below the background in certain samples can be reflecting technical problems that could be related to sample processing. By default, GUANIN flags samples that have more than 80% of the genes expressing above the background. Lower or higher % values can be used for a more stringent or relaxed filtering. Note that for some miRNA experiments a higher % of low expressed genes could be expected, so increasing this value to 90/95 % could be appropriate.
2. Samples with Fields of View (FOV), Binding density (BD), positive controls linearity or scaling factor values below or above the recommended values can be also indicating sample issues (Fig 7. Point 5 – Point 8; see NanoString Gene Expression Data Analysis Guidelines and Chilimoniuk et al. (2024)):
   - FOV default values: [0.75 – 1]. A significant difference between the expected fields of view (FOV Count) and the number of detected fields of view (FOV Counted) could suggest a technical problem. Although values below 0.75 are not recommended, the user could use samples with FOV below this threshold for downstream analysis if appropriate. Binding density default values: [0.1 – 1.8] (note: these are the recommended values for SPRINT instruments, recommended range for MAX/FLEX is 0.1 – 2.25). Overlapping probes can lead to a saturation within the image sections, resulting in significant data loss.
   - Positive controls linearity default values: [0.75 – 1]. Positive controls are used to evaluate hybridization efficiency and assay linearity. This metric performs a correlation analysis in log2 space between the known concentrations of positive control target molecules added by NanoString and the resulting counts. Correlation values lower than 0.95 may be indicative of an issue with the hybridization reaction and/or assay performance.
   - Scaling factor default values: [0.3 – 3]. Positive Control scaling factor exceeding 3-fold (0.3 – 3.0) may indicate significant under-performance of a lane or lanes. Care should be taken when interpreting results from such experiments. Extreme values for scaling factors could be typical of samples with unusually low positive control counts.

Aside from setting thresholds for QC analysis, lanes that do not meet QC requirements can be removed from the analysis, just flagged as suboptimal or manually removed (Fig 7. Point 9).

Problematic samples can be manually removed from the pipeline (Fig 7. Point 10) by directly providing GUANIN the *SampleIDs* or *filenameIDs* separated by spaces (ie. “sample1 sample2 sample3”). Consequentially, spaces within *SampleIDs* (or *filenameIDs*) are not allowed.

An initial QC using default parameters is automatically carried out once the RCC files are loaded. Then, parameters can be tuned and refined using new QC thresholds in an iterative way many times (Fig 7. Point 12). At this step, updated QC reports using the new QC parameters are generated and can be automatically opened in the default web browser (Fig 7. Point 11). Figure 8 shows an example of the filtered QC inspection report.

**miRNA assay specific QC parameters:**

If GUANIN detects the presence of ligation controls from a miRNA assay, the Quality Control tab will display 3 specific miRNA options:

- Filter by positive ligation scaling factor: Applies the same 3-log threshold used for the positive controls scaling factor, but in this case calculated from the positive ligation controls.

- POS ligation controls: checks if all positive controls are expressed (higher than background). In some cases, it might be useful to use a less restrictive threshold by filtering using the mean expression of the positive ligation probes being higher than background.

- NEG ligation controls: All negative controls counts are expected to be lower than the background threshold. Negative ligation controls with count values above background might indicate issues with ligation, potentially generating artificial counts.

Additionally, a specific miRNA QC plot will be generated showing positive and negative ligation controls with a number of counts very close to background level, in *output/images/ligplot.png.*

In this step, the following output QC files and reports are generated:

rawcounts.csv (raw matrix counts of endogenous genes)

rawfcounts.csv (raw matrix counts of filtered samples, endogenous genes)

dfhkecounts.csv (raw matrix counts of housekeeping genes)

posnegcounts.csv (raw matrix counts of positive and negative controls)

Filtered QC report in pdf format (see Figure 8).

Filtered lanes report (infolanes html file)

File containing samples flagged/discarded and reason (QCflags.txt)


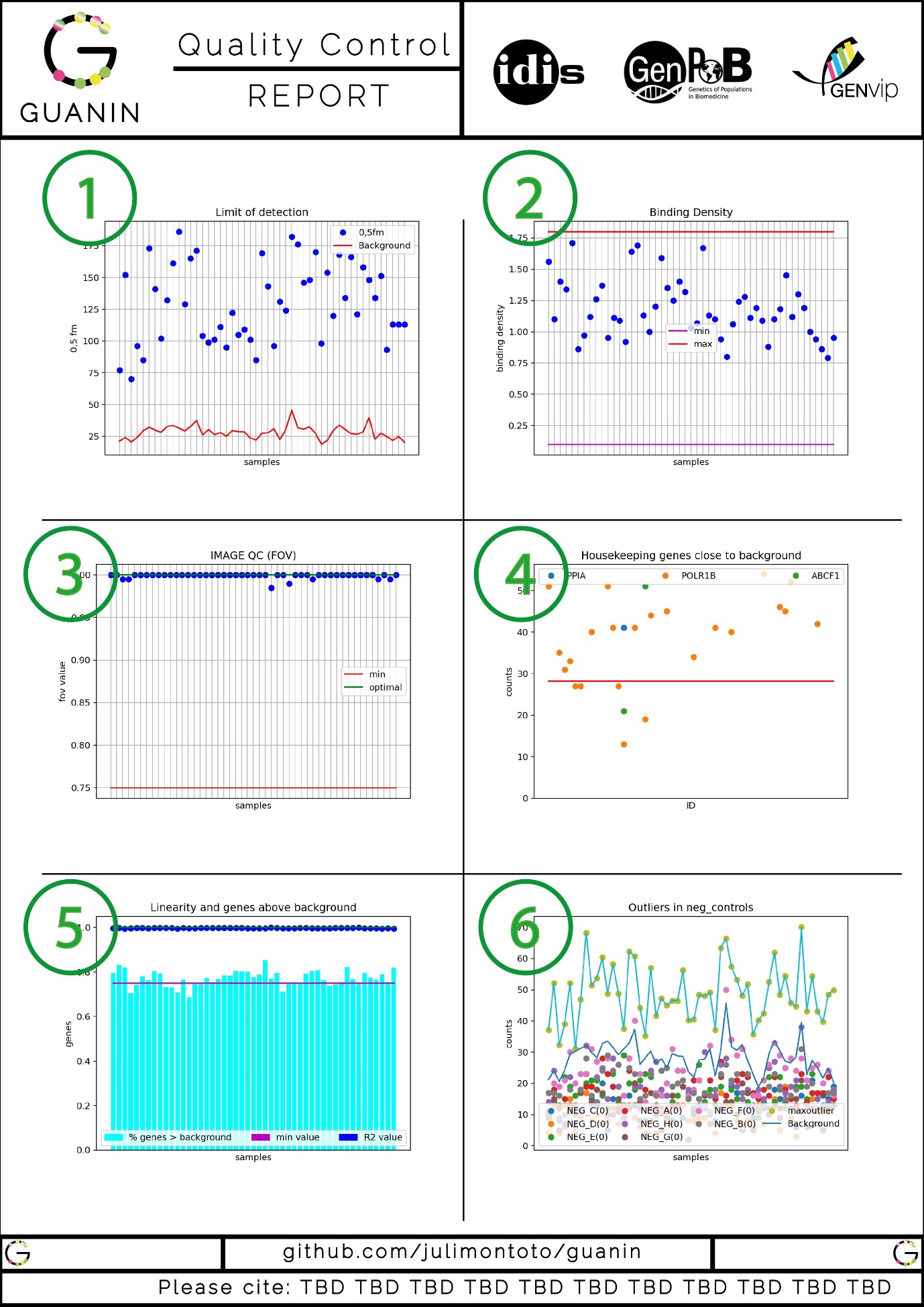


**Figure 8: pdf filtered QC report.**

**1.5 FIRST STEP OF NORMALIZATION - METHOD**

Two different methods can be chosen to perform the normalization step (Fig 9. Point 1):

A) Using scaling factors (standard procedure in *n*Solver and in most of existing tools), with extended parameterization (see Section 2.2).

B) *RUVgnorm*: Method for removing unwanted variation (see Section 2.3.1).

1. The normalization using scaling factors divides the process into 2 subprocesses, assessing technical and content normalization.

A.1) Technical normalization step using counts from the positive control probes included in the panel.


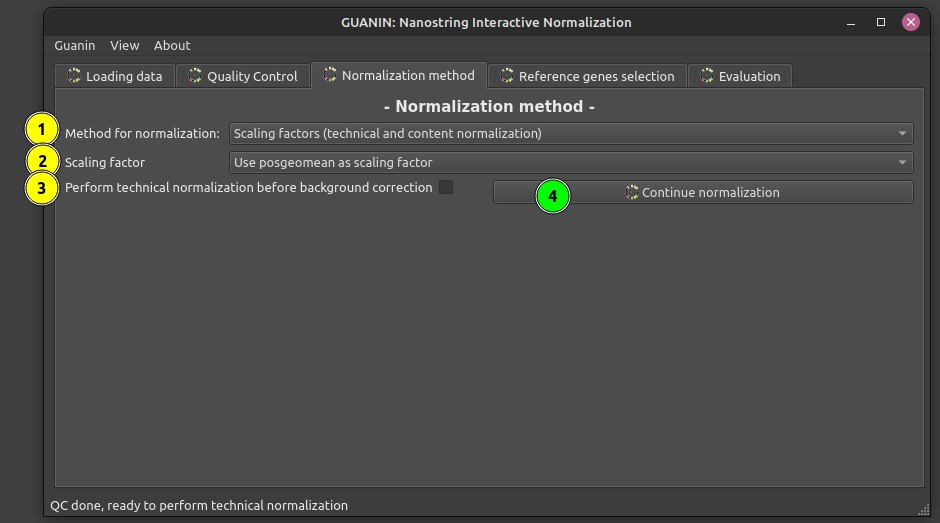


**Figure 9: GUANIN 'Normalization method' tab, using scaling factors.**

Positive controls lane-specific scaling factor (Fig 9. Point 2) can be calculated using:

1. Geometric mean of positive controls (*posgeomean*; default)
2. Sum of positive controls (see Section 2.1)
3. Median of positive controls
4. Regression: this method is based on random-coefficient hierarchical regression models and considers the expected counts of each positive control gene (see Section 2.2).

Although NanoString *n*Solver performs the background correction before the technical normalization, other tools showed better normalization performance by applying background correction after technically normalized data (see Section 2.3). This last normalization option could be also selected by the user if desired (Fig 9. Point 3).

A.2) Housekeeping normalization (continue normalization in next tab, Fig 9 Point 4).

B) *RUVgnorm*: see Section 2.3 for details on the NanoString-RUVSeq normalization procedure. Figure 10 shows the tab corresponding to the RUVgnorm method. Default k value (Fig. 10. Point 2) is set to 3, although it is recommended to iterate the process in order to find the best value that removes unwanted variation without removing biological variation. Median of ratios pre-normalization (Fig 10. Point 3) (see appendix 3.2) can be performed as it is usually part of standard RUVg normalization pipeline.


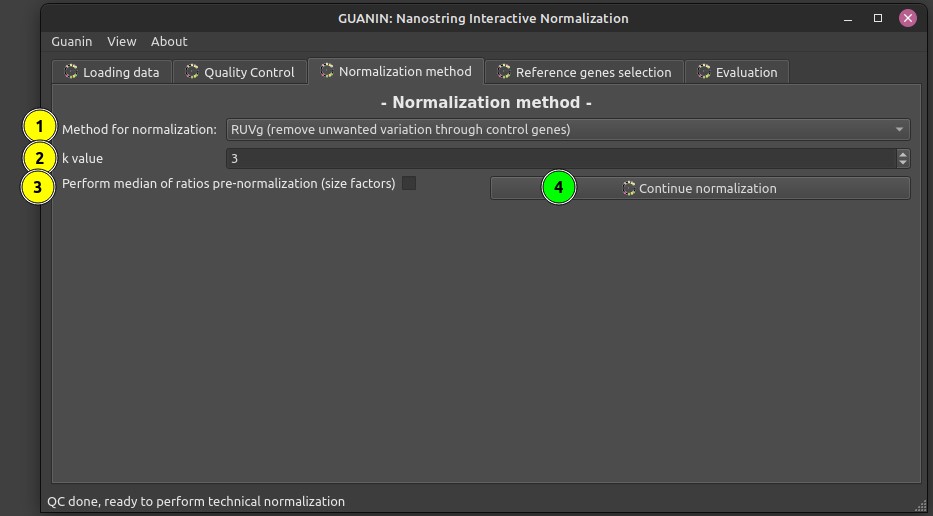


**Figure 10: GUANIN 'Normalization tab', using RUVg**

When running this step (Fig 10. Point 4), the following file is generated:

Matrix counts after the first step of normalization (tnormcounts.csv)

**1.6 SECOND STEP OF NORMALIZATION – REFERENCE GENES**


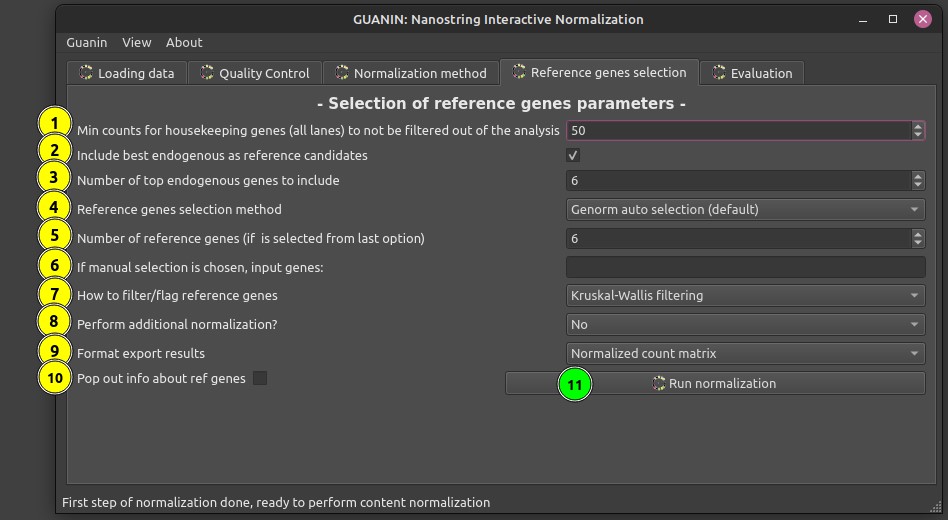


**Figure 11: GUANIN 'Selection of reference genes' tab.**

Choosing appropriate reference genes is key for a correct data normalization. GUANIN allows the user to use different methods for housekeeping and reference genes selection to normalize the data.

User can filter housekeeping panel genes and endogenous candidate reference genes by minimum counts (Fig. 11. Point 1). Setting this value to 0 would mean that no filtering is applied, while 50 is the NanoString recommended value. However, higher or lower values might be recommended for some datasets. As ideal housekeepings are those genes with a relatively stable expression above all samples, it is strongly recommended to remove those housekeepings showing low expression in any of the samples of the dataset.

GUANIN offers the novel possibility to use *ERgene.FindERG* method (see Section 2.4) to screen endogenous genes looking for additional candidate reference genes by selecting the most stable and highly expressed (Fig. 11. Point 2). This is especially useful when the housekeeping genes are poorly expressed or related to the condition. We can also manually select the number of top candidate genes found by *ERgene* to include (Fig 11. Point 3).

After the user selection (for instance filtered housekeepings + candidate endogenous genes), *geNorm* algorithm ranks the candidate genes by stability (Fig 11. Point 4) and determines which is the adequate number of them to be included as reference genes for normalization (see Section 2.5.1). User can manually select the number of reference genes (overriding *geNorm* best number of reference genes calculation) and/or which selection of reference genes from *geNorm* ranking (Figure 11. Point 5) suits better the assay. Additionally, we have developed a new feature (see Section 2.5.2) that allows to weight each gene according to its suitability (average expression stability value [M value according to *geNorm* nomenclature]); this option is especially interesting when top reference genes from geNorm are significantly better than the rest of reference genes selected.

When using scaling factors to perform content normalization, the user can also use all genes or top n expressed genes to calculate content scaling factor.

Sometimes there are some well-established reference genes that are adequate for a specific assay. The user can use all genes or top n expressed genes for content normalization. Lastly, in this case, these reference genes can be manually uploaded (separated by spaces) to use in the content normalization step (Fig 11. Point 6).

Guanin can also calculate differences in expression between the comparison groups or conditions of interest provided by the user of the reference candidates genes (Fig 11. Point 7; see Section 2.5.3). This feature uses Kruskal-Wallis/Wilcoxon statistical tests to assess statistical significance between groups and generates a report with the results (ranking_kruskal_wilcox.html). Users could filter out reference candidate genes with significantly different expression between comparison groups or conditions of interest.

Additionally, a machine learning reverse feature selection ranking report can be generated to detect which combination of reference genes are more significantly (and how much) associated with the condition (or group) of interest. Despite the result from this analysis is only informative, it might be useful to check for additive effects relating a particular set of reference gene-sets to the condition (or group) of interest. For instance, if a combination of reference genes has a high predictive value for the condition, the user could assess whether these candidate genes should be discarded as reference genes (see Section 2.5.3c).

Additional ‘normalization’ is available to format standardized output (Fig 11. Point 8). Expression in output file can be normalized count in standard units or normalized log transformation counts (rnormcounts.csv; Fig 11. Point 9). Information about reference genes and their statistically association with the condition can be automatically open in the default web browser by clicking the option in Fig 11. Point 10. Once all parameters were revised and customized by the user, normalization can be carried out (Fig 11. Point 11).

In this step, the following QC files and reports are generated:

refgenes.csv (Pre-normalized count matrix from selected reference genes).

rnormcounts.csv (Content-normalized count matrix).

*P*-values of statistical tests for individual genes between conditions or groups of interest (ranking_kruskal_wilcox.csv and .html) (see Figure 12).

Reverse feature selection metrics (Metrics_reverse_feature_seletion.html) (see Figure 13). See Section 2.5.3c for detailed information. Main columns to be focused on are *avg_score* and *feature_names* (gene names).


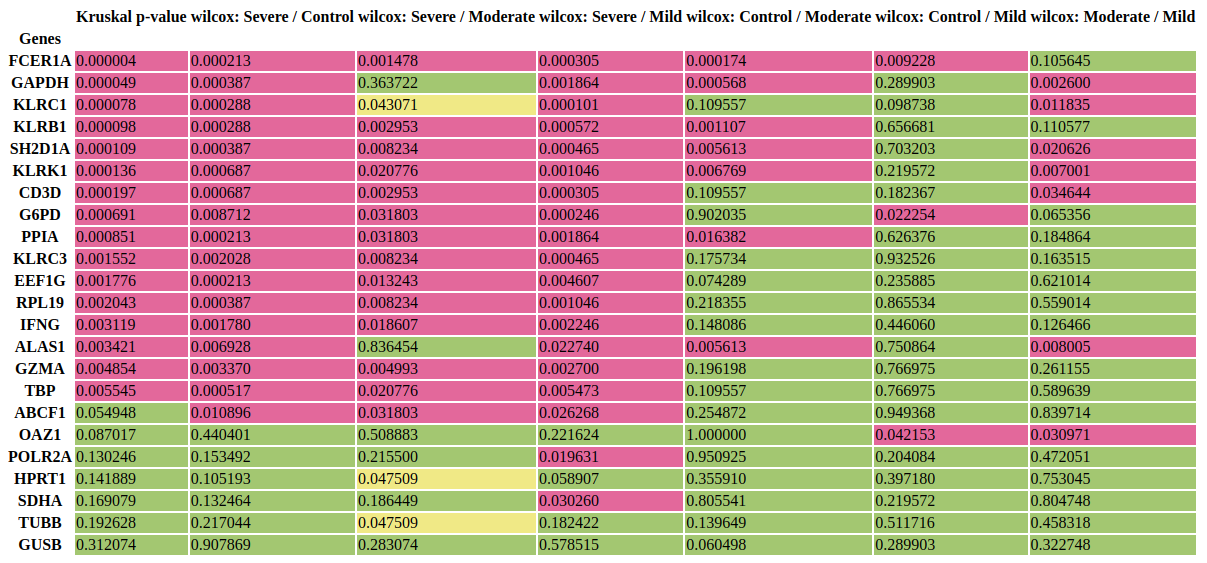


**Figure 12: P-values of statistical tests for reference genes between conditions.**


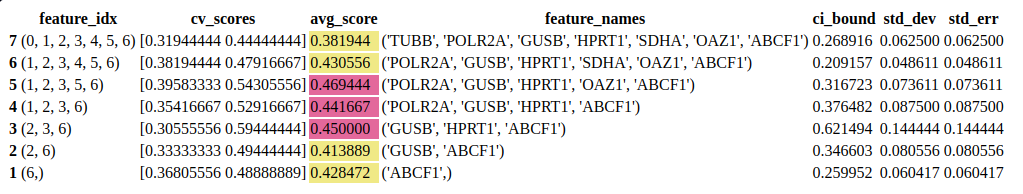


**Figure 13: Reverse feature selection metrics for candidate reference genes.**

**1.7 NORMALIZATION RESULTS**

To assess if normalization is offering reasonable results, GUANIN provides two kind of visualization tools:

a) RLE plots (Fig 14. Point 3-4), comparing pre-normalization and post-normalization data. Usually, narrower and 0-centered boxplots mean less technical variability.

b) PCA plots (Fig 14. Point 5-6), comparing how our raw and normalized matrix counts are related to batch (if provided by the user) and condition (group), in order to visualize if normalization process is effectively removing batch effect and keeping condition-related information. PCA plots can be generated by group or batch (Fig 14. Point 1).

In order to access results and intermediate files generated, in this evaluation tab the user can directly explore the output folder in file explorer (Fig 14. Point 7).


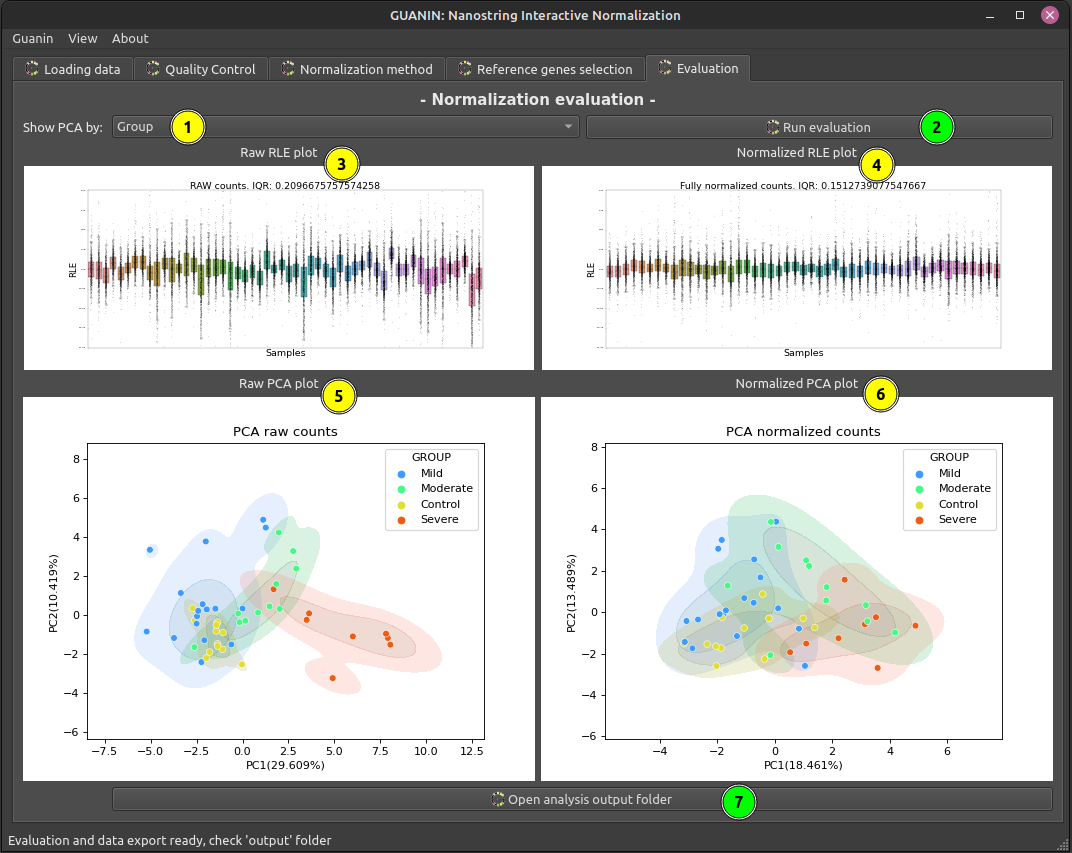
**Figure 14: GUANIN 'Normalization evaluation' tab.**

In this step, the following report is generated: norm_report.pdf (*geNorm* results, RLE plots and IQR, see Figure 15).


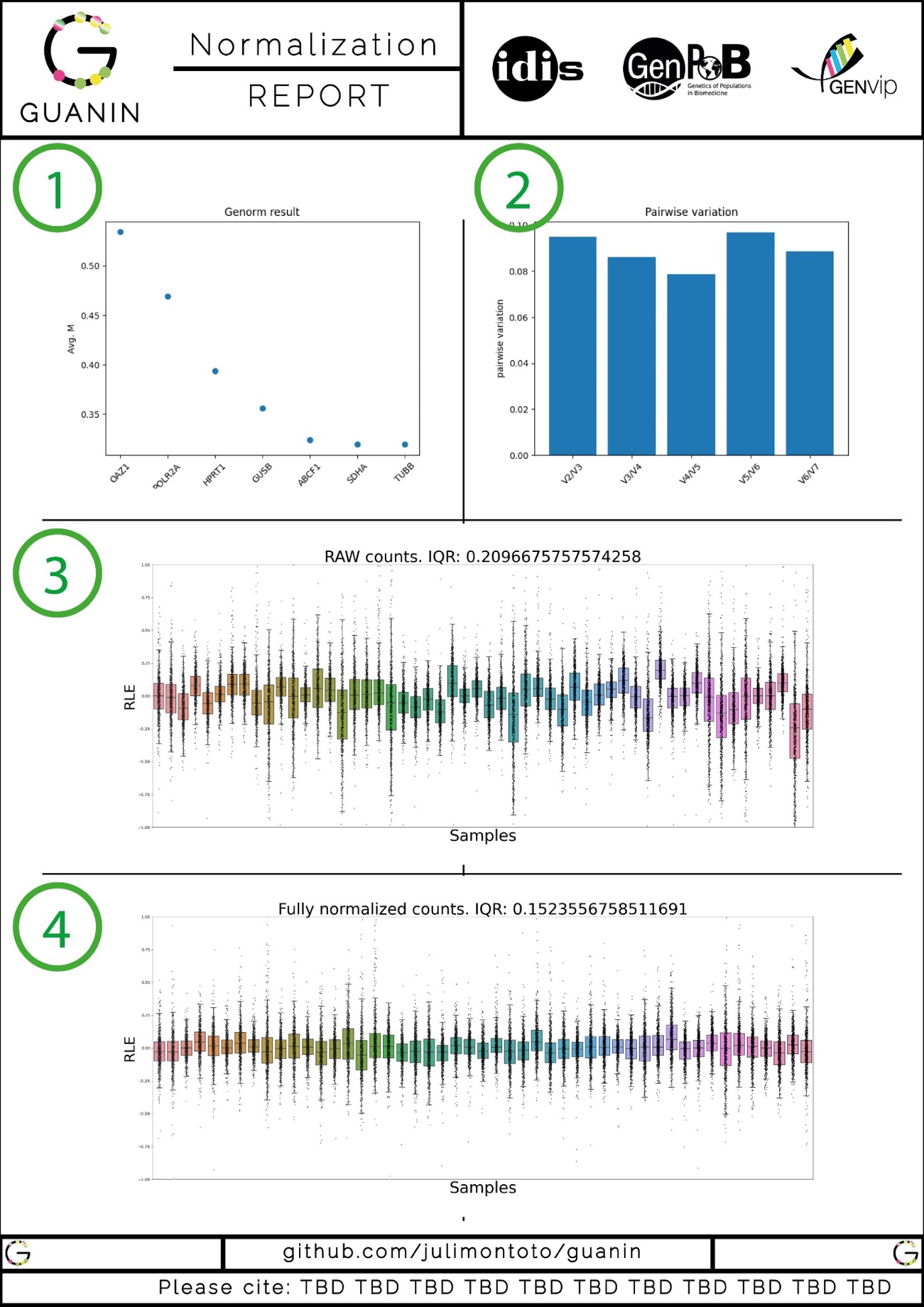


**Figure 15: Normalization report.**

Figure 15 shows an example of normalization report file. It contains 4 different plots:

1. *GeNorm* avg M value: showing average M values after stepwise exclusion of the least stable control gene. The last gene is considered the most stable reference gene.

2. *GeNorm* pairwise variation: analysis between the normalization factors NF_n_ and NF_n+1_ to determine the number of control genes required for accurate normalization (see Vandesompele, et al., 2002). for details

3/4. Relative log expression (RLE) plot for raw unnormalized data and normalized data.

**1.8 OUTPUT FILES**

Output files are generated in the default temporal path or user selected directory. Log file is always generated in temporal path and linked to the user selected directory. QC and normalization plots are stored in *images* directory, informative dataframes in .csv and .html format are stored in *info* directory. Intermediate files, including separated dataframes for different gene types and other intermediate filtering and normalization dataframes are stored in *otherfiles* directory. Reports are stored in *reports* directory and final normalized dataframe will be located in results directory.

**Images folder:**

This folder contains the following .png files:

1. avgm: Average M value for each candidate reference gene being added to geNorm selection, showing allegedly decreasing M value when adding candidate reference genes to geNorm (Vandesompele et al. 2002) selection (related to *eme.png* and *avgm.png*).

2. bdplot: Binding density values for each sample. It contains values among the minimum and maximum selected values.

3. eme: Measured M value for each gene added to the subset of candidate reference genes geNorm selection (related to *avgm.png* and *uve.png,* see (Vandesompele et al. 2002).

4. fovplot: Image QC showing the field of view values for each sample, that should be close to 1 and above the selected minimum value.

5. genbackground: Total count of genes per sample above the background.

6. hkelplot: Housekeeping genes with expression counts close to background. This image allows the user to easily check if some of the housekeeping genes are showing low expression values (or not expressed).

7. hkeplot: plot of housekeeping genes counts to visually examine the highest expressed housekeeping genes.

8. ldplot: Limit of detection plot, plotting 0.5fm value (POS_E positive control). This value is assumed to be the system’s limit of detection.

9. linplot: Linearity and genes above background plot. This plot combines these two measures for concise report matters. Bars show the same information as in genbackground.png, while dots show R2 value (linearity).

10. ocnplot: Outliers in negative controls. This plot shows counts from negative controls probes with respect to the background and the maxoutlier values (maximum expression value for considering a negative control as an outlier). Expression values for the negative control genes should be below the background.

11. pcanorm: PCA of normalized counts, grouped by batch or group (phenotype), if they have been specified by the user.

12. pcaraw: PCA of raw counts, grouped by batch or group, if they have been specified by the user.

13. rlenormplot: Relative Log Expression plot of normalized counts.

14. rlerawplot: Relative Log Expression plot of raw counts.

15. scaplot: Scaling factor per sample plot, showing the minimum and maximum values (3-fold variation range).

16. uve: Pairwise variations to determine the possible need or utility of including more than three genes for normalization. The algorithm finds a suitable number of reference genes (related to *avgm.png* and *eme.png*). See Vandesompele et al. (2002) for details on the pairwise calculations.

**Info folder:**

1. infolanes: Basic information about samples after QC filtering.

2. infolig: Basic information about QC ligation values.

3. ranking_kruskal_wilcox: Information about Kruskal-Wallis and Wilcoxon group analysis and filtering.

4. rawinfolanes: Basic information about samples before QC filtering.

5. rawsummary: Summary of infolanes before QC filtering.

6. summary: Summary of infolanes after QC filtering.

**Otherfiles folder:**

1. dfgenes: Intermediate dataframe of expression counts for the current step of the analysis. Relevant to the execution of the program.

2. dfgenes_qc: Intermediate dataframe of expression counts after applying QC filtering.

3. dfgenes_raw: Intermediate dataframe of expression counts before applying QC filtering.

4. dfhkecount: Dataframe containing housekeeping genes counts.

5. dflig: Dataframe containing ligation control genes counts.

6. dfnegcount: Dataframe containing negative control genes counts.

7. flagged: List of flagged samples by QC.

8. logarized_rnormcounts: Normalized data file, with logarithmic transformation applied (see Further Analysis section for more information).

9. posnegcounts: Dataframe of positive and negative control genes counts.

10. rawcounts: Dataframe of raw expression counts before QC filtering.

11. rawcountsf: Dataframe of raw expression counts after applying QC filtering.

12. refgenes: Dataframe of selected reference genes used for content normalization or RUVg normalization.

13. rngg: Dataframe of log counts after applying the content normalization generated for internal needs of the program (not useful for down-stream analysis; it could be not different to logarized_rnormcounts depending on the normalization step).

14. tnormcounts: Dataframe of technical normalized counts, before applying content normalization.

15. W: Dataframe of unwanted variation components calculated by RUVg algorithm, that can be used as covariables in linear regression models for differential expression analysis.

**Reports and results folders:**

1. metrics_reverse_feature_selection: Results of reverse feature selection method.

2. norm_report: Normalization report.

3. QCflags: List of samples flagged by QC analysis, reason (parameter) and value which has led to the flag.

4. QC_inspection_filtered: QC report after applying QC filtering.

5. QC_inspection: QC report before applying QC filtering.

6. rnormcounts: Resulting dataframe with normalized counts.

**1.9 FURTHER ANALYSIS**

Different available tools and methods to perform downstream differential expression analysis can be applied using the result files obtained with GUANIN:

- DESeq2 package: using the pre-normalized, QC filtered dataframe (dfgenes_qc.csv file) and including W.csv file in design formula as covariables (see Risso et al. 2014 for details). DESeq2 can be also used through the version implemented in some NanoString specific packages such as NanoStriDE.
- Limma (linear models for microarray data) package: using the normalized log counts (logarized_rnormcounts.csv file). Other tools that implement limma for differential expression analysis can be used , such as NanoTube (data can be loaded after applying QC, and “housekeeping” genes can be introduced after finding best candidate reference genes subset) or nanoR package.
- Studentś t-test approach: using R packages such as nanoR or NanoStriDE, or nSolver NanoString software, using normalized counts dataframe (rnormcounts.csv).
- Wilcoxon rank-sum test: non-parametric method. Require larger sample sizes. It requires normalized log counts (logarized_rnormcounts.csv as input file).
- Bayesian LASSO method implemented in R package RCRdiff (use QC filtered (dfgenes_qc.csv).

For more information about further analysis see Chilimoniuk et al. (2024).

A differential expression tab could be implemented in future versions of GUANIN.

**2. GUANIN METHODS**

In this section we detail features implemented in GUANIN that are ahead of the *n*Solver standard guidelines. Both brand new features introduced in GUANIN and state-of-the-art included features from different approaches and tools are explained and referenced.

2.1. ALTERNATIVE BACKGROUND SELECTION

GUANIN implements a new alternative approach to define the background, as in certain occasions default negative controls are not as stably unexpressed as it is expected. Because of this, GUANIN ranks the endogenous genes in terms of lower expression and stability among samples, and selects the 10 with less mean expression and standard deviation in order to calculate the alternative background. This alternative background is defined by the mean + twice the standard deviation of these 10 alternative negative controls.

2.2. METHODS FOR TECHNICAL NORMALIZATION

The summation of positive controls to calculate lane-specific scaling factors used in GUANIN has been already included in some state-of-the art tools as *NanoStringQCPro* (https://rdrr.io/bioc/NanoStringQCPro/).

Technical normalization by fitting a regression model uses the information of counts from positive controls and known concentration of probes to improve technical normalization (Jia, et al., 2019). A new python algorithm based on this approach has been implemented in GUANIN. It is based on NumPy’s polynomial fit and uses a least squares method to fit observed data to expected concentration of positive controls.

Although NanoString Gene Expression Data Analysis Guidelines (https://nanostring.com/wp-content/uploads/Gene_Expression_Data_Analysis_Guidelines.pdf) proposes to perform the background correction before the technical normalization, other tools, such as NACHO (Canouil, et al., 2020), implement as default method a background correction that carry out the technical normalization before background correction. This method could improve normalization in some scenarios, thus GUANIN allows choosing the order of application.

2.3. RUVg NORMALIZATION

GUANIN includes a python implementation of *RUVg* algorithm for removing unwanted variation. This new algorithm is based on the *RUVg* algorithm from *RUVSeq* R package (Risso, et al., 2014) and the method published by Bhattacharya et al. (2021). As for the size factors: the median of ratios normalization method is employed in *DESeq2* (Love, et al., 2014) to account for sequencing depth and RNA composition. A ratio between each sample and the “pseudo reference” is calculated for each gene. *DESeq2* defines size factors as the median of these ratios for each sample (median is used so any outlier genes will not affect the normalization process). *GUANIN* calculates size factors using the algorithm from *PyDESeq2* (Muzellec, et al., 2023), a python package for bulk RNA-seq differential expression analysis.

2.4. ENDOGENOUS CANDIDATES AS REFERENCE GENES

Other tools rely only in housekeeping genes to carry out the content normalization. However, sometimes housekeeping genes included in the panel are expressed below the background threshold or their expression is not stable across the samples, *GUANIN* implements the *ERgene.FindERG* method (Zeng, et al., 2020) to screen endogenous genes looking for additional candidate reference genes by selecting the most stable and highly expressed.

2.5. REFERENCE GENES SELECTION

*GeNorm* algorithm (Mestdagh, et al., 2009) is a popular algorithm to determine the most stable reference genes from a set of tested candidate reference genes in each sample panel. The use of *geNorm* algorithm in NanoString panels has been widely used (Foye, et al., 2017; Gomez-Carballa, et al., 2022). *GUANIN* uses this algorithm to get a ranked list, in order of suitability for normalization, of the candidate reference genes (housekeeping and candidate *findERG* endogenous). It also calculates the gain of information each gene adds, to set the optimal number of genes to be used as reference genes from the ranked list. During our experience with the development of *GUANIN*, we found that *geNorm* usually selects most of the endogenous included as candidates by *FindERG*, but purges most of the predefined housekeeping genes. In addition to the geNorm ranking, we introduced a pondered *geNorm* selection: Out of *geNorm* ranking, we introduced a feature that weights each gene by its *geNorm* rank and suitability (M value), and translates that score into normalization scaling factor. Genes with higher M value (calculated by *geNorm*) are weighted more in the calculation of the normalization factor. *geNorm* selects the optimal subset of genes and provides a ranking and a score, but the normalization factor is traditionally calculated only through the geometric mean of the expression of reference genes. We propose, instead, a weighted mean that considers the geNorm score to assign a weight that is used to compute the mean expression of the sample. Table 1 shows an example of pondered normalization weight calculated from *geNorm* score.

| RGN | EV | R | S | SNV | PNW |
| --- | --- | --- | --- | --- | --- |
| Gene1 | 3500 | 1 | 9.7 | 1 | 1.48 |
| Gene2 | 2500 | 2 | 9.3 | 1 | 1.43 |
| Gene3 | 2600 | 3 | 9.2 | 1 | 1.41 |
| Gene4 | 2400 | 4 | 8.8 | 1 | 1.33 |
| Gene5 | 3000 | 5 | 6.5 | 1 | 0.95 |
| Gene6 | 2200 | 6 | 5.5 | 1 | 0.83 |
| ‍Gene7 | 2300 | 7 | 5.2 | 1 | 0.81 |

**Table 1: Counts, geNorm ranking, score and normalization weights. RGN: Reference Gene Name; EV: Expression Value (counts); R: Genorm Ranking; S: Genorm Score, SNW: Standard Normalization Weight; PNW: Pondered Normalization Weight**

To check if the expression of reference genes candidates is significantly different between conditions or groups of interest, GUANIN implements 3 different methods:

a) Kruskal-Wallis test: compares two or more unmatched groups. It is applied amongst all conditions and ranks genes from the least associated with the condition and the most associated with the condition. Genes with *P*-values < 0.05 are differentially expressed between conditions and, therefore, user should assess whether include them as reference genes.

b) Wilcoxon test: for pairwise group comparisons. Genes with *P*-values < 0.05 are differentially expressed between conditions and, therefore, user should assess whether include them as reference genes.

c) Sequential Feature Selection: GUANIN incorporates a machine learning algorithm (*sklearn Sequential Feature Selector,* https://scikit-learn.org/stable/modules/generated/sklearn.feature_selection.SequentialFeatureSelector.html). It removes features (genes) to form a feature subset in a greedy fashion. At each stage, this estimator chooses the best gene to remove based on the cross-validation score of an estimator related to the prediction of the condition. The algorithm infers the condition of each sample from the expression values of a combination of reference candidate genes. If the expression of a combination of reference candidate genes is reasonably homogenous across the samples, the algorithm will not be able to precisely classify samples into the conditions (low classification accuracy). However, a combination of genes (even if they are not individually significantly associated with the condition) can be informatively enough to correctly classify the samples within conditions. The algorithm starts with the full set of candidate reference genes and sequentially removes the least informative. Optimal result for classification accuracy (low accuracy, meaning reference gene expression not related to condition) should mean *avg_score* is close to 1/j, where j is the number of groups in our experiment. For example, in an experiment with 2 conditions, if the information from those genes leads the algorithm to a 0.5 *avg_score*, it would not be more predictive than randomness. If accuracy is closer to 2*(*i*/*j*) than to 1/*j* (in the 2 conditions example, if accuracy is closer to 100% than to 50%), it could be warning that there is some cumulative information from reference genes that allow the algorithm to associate its expression to the condition. This algorithm does not allow the user to automatically filtering candidate reference genes and it is merely for informational purposes.

**3. EXAMPLE DATASETS**

We have examined 3 datasets obtained from the GEO database (https://www.ncbi.nlm.nih.gov/geo/): one in-house dataset of a COVID-19 study (GSE183071), and 2 additional datasets (GSE160208 and GSE108395) to illustrate 2 of the most common problems when preprocessing NanoString nCounter experiments (poor reference genes and poor QC) and how GUANIN can address these issues. All of them are available at Github repository of GUANIN (https://github.com/julimontoto/guanin)

**D1: GSE183071 – Blood study of gene expression profiling on the nasal epithelium in COVID-19 severity.**

This dataset consists of 54 samples including controls, mild, moderate and severe severity COVID-19 patients.
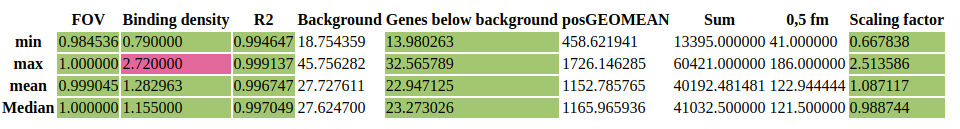
 Raw data QC output points to a problem with the binding density in one of the samples:

***Figure 16: Infolanes summary for preprocessing QC.***

After checking the *infolanes* file, high binding density values can be observed for 5 samples (B0513, B0318, B0319, B0369, B0343).

User can select an alternative max binding density threshold to keep these samples since the default threshold value will discard them for downstream analysis. After filtering out these 5 samples, all samples successfully passed the QC thresholds.

In this example, we are using scaling factors for content normalization, using *posgeomean* as scaling factor for technical normalization and background correction was performed before technical normalization. To check the suitability of the housekeeping genes included in the panel, we carried out the analysis only with these housekeepings. We can see that many (10/14) of the housekeepings genes are related with some of the conditions (Fig. 17).


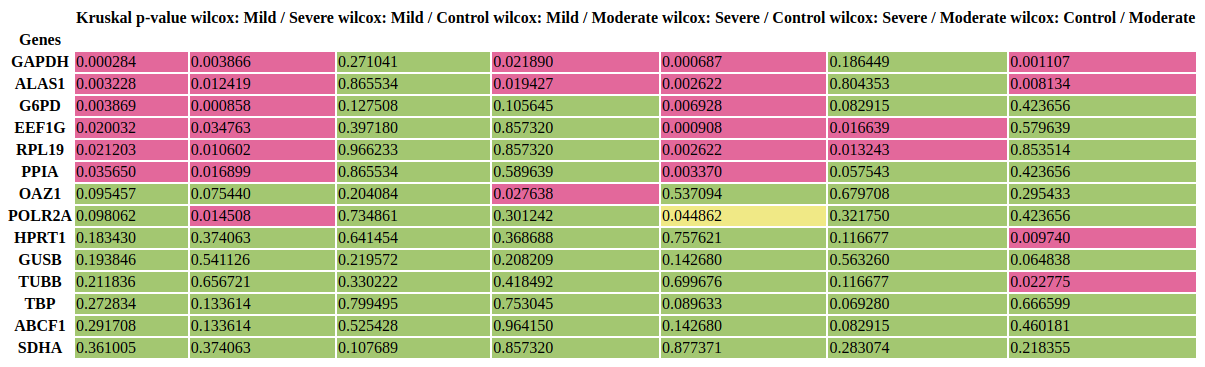


***Figure 17: Kruskal-Wallis association test between conditions for housekeeping genes.***

Then, we can try to include best endogenous genes as candidate reference genes. For this example, we have included the best 12 endogenous candidates. With this new gene subselection, we found that the Kruskal-Wallis test finds non-association with the condition for 15/24 genes, while Wilcoxon’s test is passed for 7 of them (Fig 18).


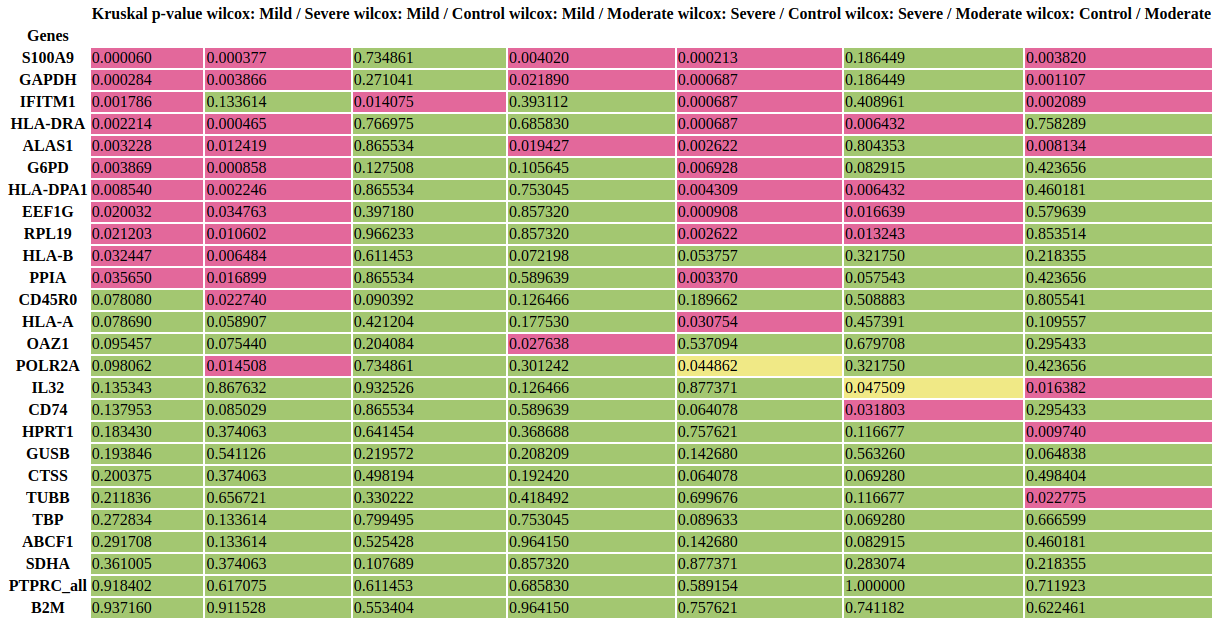


***Figure 18: Kruskal-Wallis association test between conditions for candidate reference genes.***

Assuming 7 genes might be a low number for the *geNorm* algorithm, we proceed with the analysis using the Kruskal-Wallis filtering.

Aside from this, in part probably by some relation found by Wilcoxon test, the reverse feature selection algorithm can classify condition groups with more than double the accuracy expected by randomness, which may be related to still a suboptimal selection of reference genes (Fig. 19).


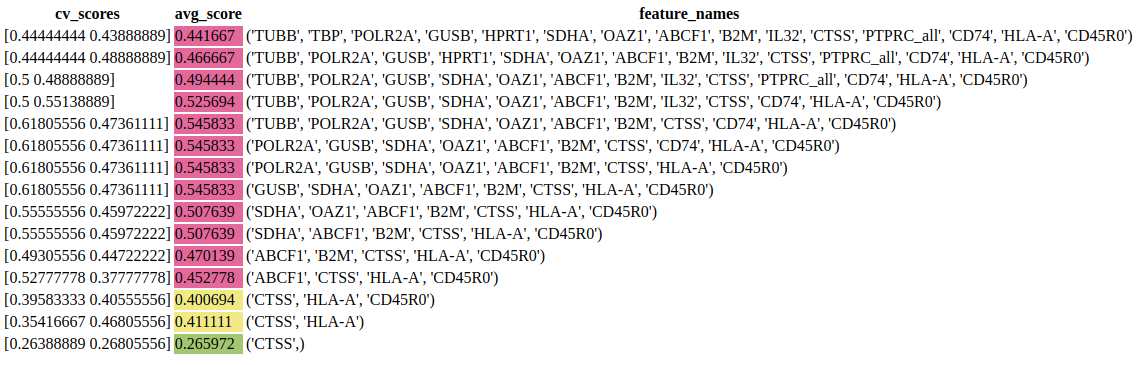


***Figure 19: Reverse feature selection algorithm results for non-filtered subset of reference genes.***

Proceeding with the analysis with Wilcoxon filtering would derive on a smaller but better sub-selection of reference genes (Fig. 20).


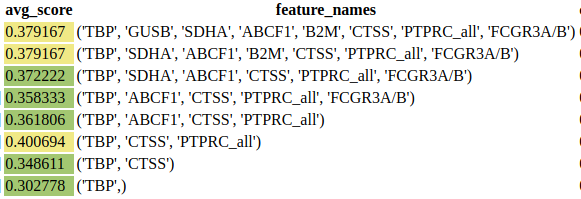


***Figure 20: Reverse feature selection algorithm results for Wilcoxon-test filtered reference genes.***

When evaluating normalization results, we notice RLE plots derived from scaling factors method center means but don’t narrow boxplots. In the other hand, PCA plots shows 3 clearly different groups. (see Figure 21, right)

Using other approaches, such as *RUVgnorm*, results offer narrower RLE plots but less separated by condition PCA plots. (see Figure 21, left).


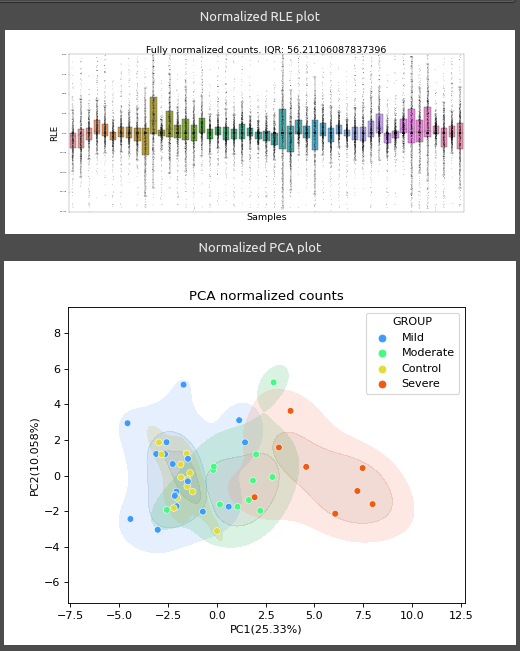


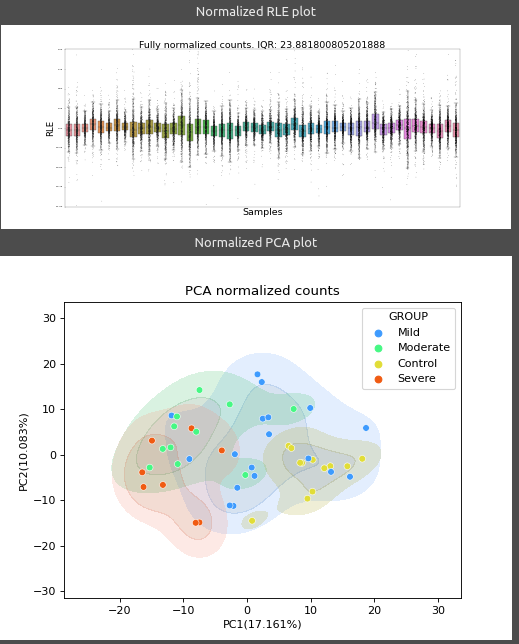


***Figure 21: Evaluation plots for dataset 1 normalization. RUVgnorm method (left) vs scaling factors method (right).***

At this point, GUANIN offers the researcher the capability to use the normalization method that better suits the experiment.

**D2: GSE160208 – Gene expression in the brain of sporadic Creutzfeldt-Jakob disease patients (CJD), and normal controls (CT).**

This dataset contains 47 samples from 2 groups: disease and control. For this dataset all samples passed QC, thus we proceed with normalization.

The results on evaluation of reference genes (panel housekeeping and best endogenous) provides only 2 genes suitable to be reference genes for content normalization. As a minimum of 3 genes are required, we have several options:

- Lower the threshold of min counts for housekeeping in case there are low but stably expressed ones that can be rescued.

- Include more best endogenous as candidates.


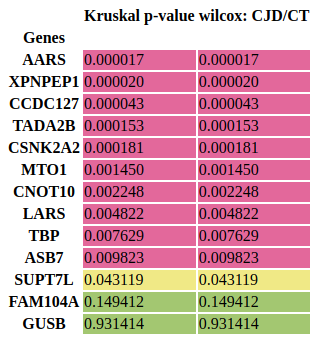

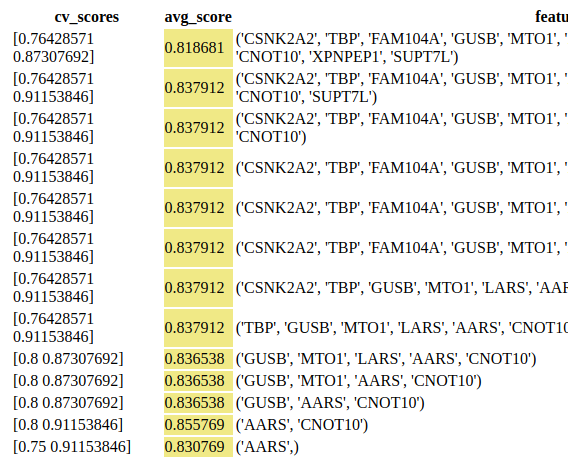
- Continue without filtering assuming reference genes are suboptimal.

***Figure 22: Kruskal and Wilcoxon test (left) and reverse feature selection algorithm (right) for candidate selection of reference genes.***

We decided to include 24 of the best endogenous genes instead of 6. Doing so, we obtain 1 more gene to be selected by *geNorm* as a suitable reference gene. Then, *geNorm* can perform the normalization using 3 reference genes: *KDM5D*, *CD24* and *EGR1*. Once this limitation with default housekeeping genes is solved, analysis can continue.

**D3: GSE160208 – Gene expression in the brain of sporadic Creutzfeldt-Jakob disease patients (CJD), and normal controls (CT).**

This dataset contains 233 samples from 2 groups: disease and control.
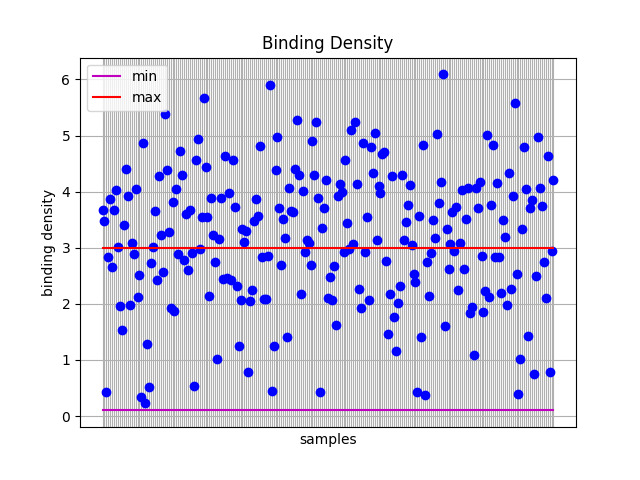

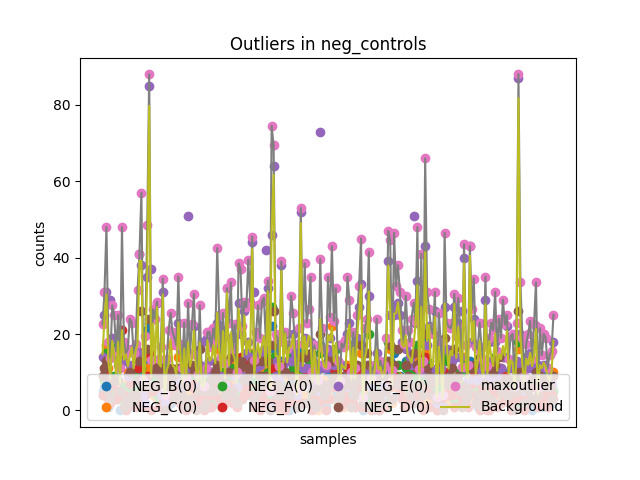
This dataset, as many others found in databases, has a lot of QC problems that would be more difficult to address with other normalization tools. We can see in the QC report that a lot of samples are above max binding density, some of them below limit of detection, one has very low *FOV* value, there are several housekeeping genes close and below the background and there also are some outliers in negative controls (Fig. 23).

***Figure 23: Alarming QC values in first inspection. Many samples with a high binding density value (left) and some negative controls being expressed (right)***

Discarding and repeating the experiment could be a reasonable choice, as only 14 samples pass QC. Another reasonable approach could be continuing the analysis with a maximum value of binding density of 3 (assuming the responsibility that this is discouraged by NanoString). We also detect that there are outliers in the negative controls, so we selected “alternative background” as background calculation method.

With this QC parametrization, we have 81 samples passing this specific QC values in the analysis.

Also, as some of housekeeping genes are not expressed (only 5 are expressed), we will refine them with a selection of the most suitable endogenous. Kruskal-Wallis test discards none of the 11 preselected genes for being associated with the condition, so we let *geNorm* algorithm to choose how many and which genes are most suitable for the analysis. From these 11 preselected genes, it selects 3 as reference genes.

RLE plots are suboptimal and PCA shows overlapping between the two conditions. This could be consequence of the poor QC that has been noted during the analysis.

GUANIN offers the researcher the possibility to detect thesese QC problems and choose to adapt preprocessing parameters in order to minimize them, or discard further analysis within the experiment at any point of the preprocessing step.

**5. CLI COMMANDS HELP**

'-f', '--folder', type=str, default= pathlib.Path.cwd() / '../examples/d1_COV_GSE183071', help='relative folder where RCC set is located'

'-minf', '--minfov', type=float, default=0.75, help='set manually min fov for QC'

'-maxf', '--maxfov', type=float, default=1, help='set manually max fov for QC'

'-minbd', '--minbd', type=float, default=0.1, help='set manually min binding density for QC'

'-maxbd', '--maxbd', type=float, default=1.8, help='set manually max binding density for QC'

'-minlin', '--minlin', type=float, default=0.75, help='set manually min linearity for QC'

'-maxlin', '--maxlin', type=float, default=1, help='set manually max linearity for QC'

'-minscaf', '--minscalingfactor', type=float, default=0.3, help='set manually min scaling factor for QC'

'-maxscaf', '--maxscalingfactor', type=float, default=3, help='set manually max scaling factor for QC'

'-swbrrq', '--showbrowserrawqc', type=bool, default=False, help='pops up infolanes and qc summary'

'-swbrq', '--showbrowserqc', type=bool, default=False, help='pops up infolanes and qc summary'

'-swbrcn', '--showbrowsercnorm', type=bool, default=False, help='pops up infolanes and qc summary'

'-lc', '--lowcounts', type=str, default='skip', choices=['skip', 'asim', 'subtract'], help='what to do with counts below background?'

'-mi', '--modeid', type=str, default='filename', choices=['sampleID','filename', 'id+filename'], help='choose sample identifier. sampleID: optimal if assigned in rccs. filenames: easier to be unique. id+filename: care with group assignment coherence'

'-mv', '--modeview', type=str, default='view', choices=['justrun', 'view'], help='choose if plot graphs or just run calculations'

'-tnm', '--tecnormeth', type=str, default='posgeomean', choices=['posgeomean','Sum', 'Median', 'regression'], help='choose method for technical normalization'

'-reg', '--refendgenes', type=str, default= 'endhkes', choices=['hkes', 'endhkes'], help='choose refgenes, housekeeping, or hkes and endogenous'

'-re', '--remove', type=str, nargs='+', default=None, help='lanes to be removed from the analysis'

'-bg', '--background', type=str, default= 'Background', choices=['Background', 'Background2', 'Background3', 'Backgroundalt', 'Manual'], help='choose background: b1=meancneg+(2*std), b2=maxcneg, b3=meancneg, balt=uses alternative subset of negative controls'

'-pbb', '--pbelowbackground', type=int, default=85, help='if more than %bb genes are below background, sample gets removed from analysis'

'-mbg', '--manualbackground', type=float, default=None, help='set manually background'

'-crg', '--chooserefgenes', type=list, nargs='+', default = None, help = 'list of strings like. choose manualy reference genes to use over decided-by-program ones'

'-fgv', '--filtergroupvariation', type=str, default='filterkrus', choices=['filterkrus', 'filterwilcox', 'flagkrus', 'flagwilcox', 'nofilter'], help='¿filter or flag preselected ref genes by significative group-driven differences? needs groups to be declared'

'-fsn', '--featureselectionneighbors', type=float, default=4, help='number of neighbors for feature selection analysis of refgenes. recommended 3-6'

'-g', '--groups', type=str, default='yes', choices=['yes','no'], help='defining groups for kruskal/wilcox/fs analysis?'

'-ne', '--numend', type=int, default=6, help='number of endogenous to find by ERgene to include in analysis to check viability as refgenes'

'-ar', '--autorename', type=bool, default=False, help='turn on when sample IDs are not unique, be careful on sample identification detail'

'-cn', '--contnorm', type=str, default='refgenes', choices=['ponderaterefgenes', 'refgenes', 'all', 'topn']

'-an', '--adnormalization', type=str, default='no', choices=['no', 'standarization'], help='perform additional normalization? standarization available'

'-tn', '--topngenestocontnorm', type=int, default=100, help='set n genes to compute for calculating norm factor from top n expressed endogenous genes'

'-mch', '--mincounthkes', type=int, default=80, help='set n min counts to filter hkes candidate as refgenes'

'-nrg', '--nrefgenes', type=int, default=None, help='set n refgenes to use, overwriting geNorm calculation'

'-lr', '--laneremover', type=bool, default=True, choices=[True, False], help='option to perform analysis with all lanes if set to no'

'-lo', '--logarizedoutput', type=str, default='no', choices=['2', '10', 'no'], help='want normed output to be logarized? in what logbase?'

'-le', '--logarizeforeval', type=str, default='10', choices=['2', '10', 'no'], help= 'logarithm base for RLE calculations'

'-gf', '--groupsfile', type=str, default='../examples/groups_d1_COV_GSE183071.csv', help='enter file name where groups are defined'

'-ftl', '--tnormbeforebackgcorr', type=int, default=1, help='0= False, 1= True, 2= ruvg'

'-of', '--outputfolder', type=str, default=tempfile.gettempdir() + '/guanin_output'

'-sll', '--showlastlog', type=bool, default = False

'-k', '--kvalue', type=int, default=3

'-pip', '--pipeline', type=str, default='ruvgnorm', choices=['ruvgnorm', 'scalingfactors']

'-wrg', '--whatrefgenes', type=list, default=[ ]

'-gpca', '--grouppca', type=str, default='GROUP'

'-dm', '--deseq2_mor', type=bool, default=True

'-mr', '--manual_remove', type=bool, default=False

'-nbl', '--nbadlanes', type=str, default='No badlanes detected'

'-bl', '--badlanes', type=set, default=set()

'-e', '--elapsed', type=float, default=0.0

'-pb', '--pcaby', type=str, default='group'

'-miR', '--miRNAassay', type=bool, default=False

'-ls', '--apply_ligscaf', type=bool, default=False

'-plq', '--posligqc', type=str, default='min_neglig'

'-nlq', '--negligqc', type=str, default='max_neglig'

'-gs', '--generatesvgs', type=bool, default=False

**References**

Bhattacharya, A., et al. An approach for normalization and quality control for NanoString RNA expression data. Briefings in bioinformatics 2021;22(3).

Canouil, M., et al. NACHO: an R package for quality control of NanoString nCounter data. Bioinformatics 2020;36(3):970-971.

Chilimoniuk J, Erol A, Rodiger S, Burdukiewicz M. Challenges and opportunities in processing NanoString nCounter data. Comput Struct Biotechnol J 2024; 23:1951-8.

Foye, C., et al. Comparison of miRNA quantitation by Nanostring in serum and plasma samples. PLoS One 2017;12(12):e0189165.

Gomez-Carballa, A., et al. A multi-tissue study of immune gene expression profiling highlights the key role of the nasal epithelium in COVID-19 severity. Environ Res 2022;210:112890.

Jia, G., et al. RCRnorm: An integrated system of random-coefficient hierarchical regression models for normalizing NanoString nCounter data. Ann Appl Stat 2019;13(3):1617-1647.

Love, M.I., Huber, W. and Anders, S. Moderated estimation of fold change and dispersion for RNA-seq data with DESeq2. Genome Biol. 2014;15(12):550.

Mestdagh, P., et al. A novel and universal method for microRNA RT-qPCR data normalization. Genome biology 2009;10(6):R64.

Muzellec, B., et al. PyDESeq2: a python package for bulk RNA-seq differential expression analysis. Bioinformatics 2023;39(9).

Risso, D., et al. Normalization of RNA-seq data using factor analysis of control genes or samples. Nature biotechnology 2014;32(9):896-902.

Vandesompele, J., et al. Accurate normalization of real-time quantitative RT-PCR data by geometric averaging of multiple internal control genes. Genome biology 2002;3(7):RESEARCH0034.

Zeng, Z., et al. ERgene: Python library for screening endogenous reference genes. Scientific reports 2020;10(1):18557.
